# The Efficacy of Pediatric‐Inspired Regimens vs. Hyper‐CVAD in the Treatment of Adolescents and Young Adults With Acute Lymphoblastic Leukemia: A Systematic Review and Meta‐Analysis

**DOI:** 10.1002/ajh.27607

**Published:** 2025-02-13

**Authors:** Wenqing Su, Melisa Stricherz, Alison Martin, Jonathan Belsey, Eric Kemadjou, Daniel J. DeAngelo

**Affiliations:** ^1^ Jazz Pharmaceuticals Philadelphia Pennsylvania USA; ^2^ Jazz Pharmaceuticals Palo Alto California USA; ^3^ Crystallise Ltd, Stanford‐le‐Hope Essex UK; ^4^ JB Medical Ltd Suffolk UK; ^5^ Department of Medical Oncology Dana‐Farber Cancer Institute Boston Massachusetts USA

**Keywords:** acute lymphoblastic leukemia, asparaginase, hyper‐CVAD, lymphoblastic lymphoma, pediatric‐inspired regimens

## Abstract

Adults with acute lymphoblastic leukemia/lymphoblastic lymphoma (ALL/LBL) have poorer outcomes than pediatric patients. The aim of this systematic literature review and meta‐analysis was to compare asparaginase (ASP)‐containing pediatric‐inspired regimens (PIRs) with hyper‐fractionated cyclophosphamide, vincristine, doxorubicin, and dexamethasone (hyper‐CVAD) in adolescents and young adults (AYAs) with ALL/LBL. Searches included relevant publications up to April 21, 2022. A meta‐analysis was conducted in four studies with comparable demographics, to estimate the effects of intervention on rates of response and survival. Patients receiving PIRs were approximately twice as likely to achieve complete response and 1.8 times more likely to survive than patients receiving hyper‐CVAD. These results suggest ASP‐containing PIRs are associated with improved outcomes in AYAs compared with hyper‐CVAD.

## 1 Introduction

Acute lymphoblastic leukemia/lymphoblastic lymphoma (ALL/LBL) are rare, aggressive hematologic malignancies most commonly seen in children [1]. The past few decades have seen remarkable improvements in the treatment of pediatric patients with ALL/LBL, including an overall long‐term survival of approximately 90%; however, outcomes in adult patients have historically lagged behind [1, 2, 3]. Although up to 90% of adults with ALL/LBL achieve complete response (CR), less than 45% of adult patients maintain long‐term disease‐free survival (DFS) [1]. Poor outcomes have been attributed to biological, treatment‐related, and psychosocial factors [4, 5, 6, 7].

Multiple studies and review articles in adolescents and young adults (AYA) with ALL/LBL show that treatment with pediatric‐inspired regimens (PIRs) is associated with improved response rate and survival outcomes compared with traditional adult protocols, prompting further evaluation of PIRs in AYA and older adult populations [4, 5, 8, 9, 10, 11, 12]. A large, prospective, multicenter study published in 2015 assessed the Dana‐Farber Cancer Institute Pediatric Consortium protocol in newly diagnosed adult patients with ALL (aged 18–50 years old) [5]. Of 92 eligible patients, 78 (85%; 90% confidence interval [CI]: 77%, 91%) achieved a CR at the end of induction. The 4‐year overall survival ([OS] 95% CI) for all 92 patients and 4‐year DFS (95% CI) for the 78 patients achieving CR were 67% (56%, 76%) and 69% (56%, 78%), respectively [5]. From 2008 to 2014, the Nordic Society of Pediatric Hematology and Oncology ALL2008 protocol recruited 1509 patients with newly diagnosed B‐cell or T‐cell ALL (aged 1–45 years) [11]. Of these, 1314 (87%) patients achieved continuous CR. The projected 5‐year OS and 5‐year event‐free survival (EFS) were 91% ± 1% and 85% ± 1%, respectively [11]. Following this, Cancer and Leukemia Group B (CALGB) 10403 [8] was a large prospective, multicenter clinical trial in North America that demonstrated the feasibility and efficacy of a PIR in AYA patients aged 17–39 years. Of the 295 PIR‐treated AYA patients with newly diagnosed B‐ or T‐cell ALL, 263 (89%; 95% CI: 85%, 92%) achieved CR at the end of induction or extended induction. The estimated 3‐year OS (95% CI) and DFS (95% CI) for these patients were 73% (68%, 78%) and 66% (60%, 72%), respectively, compared with a historical rate of 58% (52%, 64%) and 48% (41%, 55%), respectively [8].

PIRs involve high cumulative doses of vincristine, glucocorticoids, and asparaginase (ASP), with most regimens adding an anthracycline (usually doxorubicin or daunorubicin), along with intensive and prolonged central nervous system prophylaxis [13]. ASP is an enzyme that catalyzes the conversion of the amino acid L‐asparagine into aspartic acid and ammonia [14]. Leukemic cells have a reduced ability to synthesize asparagine, and therefore are dependent on an exogenous source of asparagine for survival [15]. ASP can also hydrolyze glutamine to produce glutamic acid and ammonia, with cytotoxic effects [15]. However, the primary pharmacological effect of ASP is based on the killing of leukemic cells due to the depletion of plasma asparagine [15]. In contrast to PIRs, traditional adult protocols employ high doses of cytotoxic agents (e.g., anthracycline and cyclophosphamide), typically no ASP, and more frequently involve allogenic hematopoietic cell transplantation [16]. An example of a non‐ASP‐containing regimen is hyper‐fractionated cyclophosphamide, vincristine, doxorubicin, and dexamethasone (hyper‐CVAD), a dose‐intensive therapy alternating eight total courses of fractionated CVAD with high‐dose methotrexate (MTX) and cytarabine [17]. Maintenance therapy consists of a combination of 6‐mercaptopurine, vincristine, MTX, and steroid pulses [17]. Treatment with hyper‐CVAD typically results in high CR rates (92%–95%) but low‐to‐moderate OS (38%–58% based on 5‐year and 3‐year survival rates, respectively) [18, 19].

To date, there are no prospective randomized controlled trials (RCTs) comparing PIRs with hyper‐CVAD in AYA or older adult patients with ALL/LBL. Here, we report the findings of a systematic literature review (SLR) and meta‐analysis comparing ASP‐containing PIRs with hyper‐CVAD in the treatment of ALL/LBL in AYA populations from non‐randomized, comparative studies.

## 2 Methods

### 2.1 Systematic Literature Review

We performed an SLR (using databases including PubMed, Embase, and Cochrane Library, two clinical trial databases, and eight gray literature sites) to identify relevant manuscripts and conference abstracts comparing ASP‐containing PIRs and hyper‐CVAD in patients with ALL/LBL published up to April 21, 2022. The search strategy consisted of title/abstract keywords and subject headings describing key concepts of “acute lymphoblastic leukemia,” “lymphoblastic lymphoma,” “asparaginase,” “hyper‐CVAD,” “response,” “survival,” “refractory,” and “minimal residual disease.” The detailed search strategy can be found in *Appendix A* (Tables S2–S5).

Abstracts were screened independently by two researchers (with any discrepancies resolved by the project lead), and two senior researchers conducted the full‐text review for eligibility. The inclusion and exclusion criteria can be found in *Appendix A* (Table S6). Data extraction was performed on all selected articles using Microsoft Excel, and values were extracted using the online tool WebPlotDigitizer (version 4.5) when data were only presented in figures [20]. Data synthesis categorized treatments into ASP‐containing PIRs and hyper‐CVAD for analysis. ASP‐containing PIRs were studies that included a regimen containing ASP in at least one phase of treatment, whereas studies categorized as hyper‐CVAD were studies that included a regimen consisting of CVAD.

The quality of the texts was assessed using the Cochrane risk‐of‐bias (RoB2) tool [21] for the RCTs and the Downs and Black criteria for the comparative nonrandomized studies [22]. The Cochrane RoB2 reports a categorical response, whereas the Downs and Black score is numerical. Thus, the Downs and Black scores were arbitrarily categorized into high (score 0–13), moderate (score 14–18), and low (score 19–25) risk of bias to translate the scores to equivalent categories. Two researchers cross‐checked the data extraction and bias scoring to ensure accuracy, and disagreements were resolved by discussion until reaching a consensus.

### 2.2 Meta‐Analysis

A meta‐analysis was conducted to estimate the effects of intervention on CR and OS, two commonly reported outcomes across studies identified by the SLR. From the identified primary studies, a second algorithm was applied to studies selected for inclusion in the meta‐analysis. These studies (1) compared ASP‐containing PIRs and hyper‐CVAD, (2) involved ASP treatment given during the induction or consolidation phase, and (3) did not include a mixed population of patients with the presence (ph+) and the absence (ph‐) of Philadelphia chromosome.

### 2.3 Statistical Analyses

We calculated the odds ratio (OR) for CR and the hazard ratio (HR) for OS. Among the studies selected for the meta‐analysis, only one [23] reported the HR along with the 95% CI for OS. Two studies [24, 25] reported the median OS, and one [26] reported the 5‐year OS rate. For studies that only reported median survival, the HR was estimated by assuming a constant hazard rate over time for OS within each treatment arm. It was also assumed that the OS curves followed an exponential distribution.

The meta‐analysis was conducted using both fixed‐ and random‐effects models in the R software Meta package (version 4.18‐1) [27] with a significance level of 0.05. The inverse‐variance method was used to estimate the pooled OR and HR along with their 95% CIs. Uncertainty was captured using both 95% CIs and 95% prediction intervals (PIs), which capture separate aspects of uncertainty. The level of heterogeneity across the studies was evaluated using the *I* ^2^‐statistic [28]. A scenario analysis included studies meeting the assumption of proportionality of the HR to identify potential sources of heterogeneity and bias among the studies.

One study [25] included in the meta‐analysis reported outcomes for two PIR arms compared with hyper‐CVAD. Patients in these arms were separately compared with patients in the hyper‐CVAD arm during meta‐analysis.

## 3 Results

### 3.1 Results of the SLR

**FIGURE 1:**
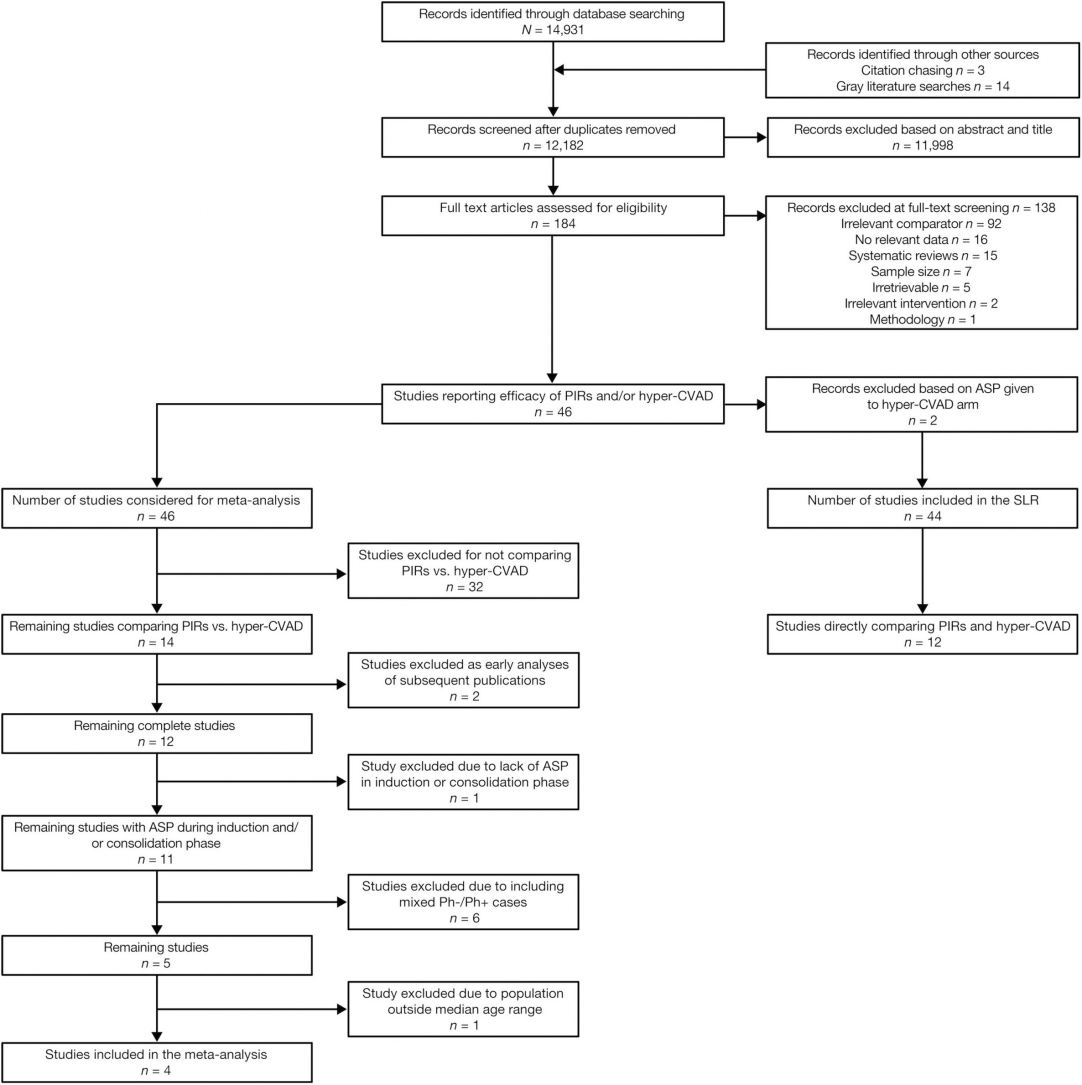
Flow diagram for selection of SLR and meta‐analysis studies. ASP, asparaginase; hyper‐CVAD, hyper‐fractionated cyclophosphamide, vincristine, doxorubicin, and dexamethasone; Ph‐/Ph+, Philadelphia chromosome absent/present; PIR, pediatric‐inspired regimen; SLR, systematic literature review.

The initial search identified 12,182 unique publications (Figure 1). Based on title and abstract screening, full texts were retrieved for 184 abstracts, three of which were identified during citation chasing, and assessed for eligibility. Of the 184 full texts that were screened, 138 were excluded. Most exclusions were due to irrelevant comparators (*n* = 92), missing relevant data (*n* = 16), or were systematic reviews (*n* = 15, Figure 1, Table S7). Ultimately, 46 primary publications of potential relevance were selected for review (Figure 1). Of these, 22 were RCTs and 22 were nonrandomized comparative studies. Direct comparisons of PIRs and hyper‐CVAD were reported in 12 studies, but none were RCTs, and all had a moderate or high risk of bias (Figure 1 and Table 1). All but three studies compared a single PIR to hyper‐CVAD (Table S1). Of these three, one had a combined‐PIR arm where patients were treated with either a Berlin‐Frankfurt‐Münster (BFM) or CALGB regimen [29]. The other two studies included separate arms evaluating BFM or CALGB regimens [25, 30]. The patients' clinical characteristics were reported inconsistently, but the demographic characteristics indicate that the median age of the study population ranged from 20 to 42 years.

**TABLE 1:** Summary of characteristics from comparative studies of ASP‐containing PIRs and hyper‐CVAD.

Study *ROB* ^*a*^	Arm	*N*	Data inclusion dates	Median duration of follow‐up	Age (years, mean/median/% < 21 years)^b^	Sex (% female)	Phenotype (% B‐cell)	CD20 expression (%)	RTX use (%)	WBC (subgroup: %)	High risk (%)	CNS involvement (%)	Included in meta‐analysis
Abbasi [35] *Moderate ROB*	CALGB 8811 (PIR)	42	Jan 2007–Dec 2011	32 months	34	38	—	—	—	< 30 × 10^9^/L: 78.6 30–100 × 10^9^/L: 14.3 > 100 × 10^9^/L: 7.1	—	11.9	No
	Hyper‐CVAD	66			32.5	36	—	—	—	< 30 × 10^9^/L: 72.7 30–100 × 10^9^/L: 18.2 > 100 × 10^9^/L: 9.1	—	9.1	
Alabdulwahab [32] *High ROB*	DFCP (PIR)	43	Mid 2011–Nov 2016	29 months	< 21 years = 72.1% ≥ 21 years = 27.9%	34.9	86	34.3	—	< 30 × 10^9^/L: 62.8 ≥ 30 × 10^9^/L: 37.2	37.2^c^	—	No
	Hyper‐CVAD	30			< 21 years = 16.7% ≥ 21 years = 83.3%	36.7	50	53.3	0	< 30 × 10^9^/L: 56.7 ≥ 30 × 10^9^/L: 43.3	33.3^c^	—	
Alacacioglu [36] *Moderate ROB*	BFM (PIR)	20	Mar 2006–Oct 2012	37 months	25	25	80	—	—	—	60^d^	5	No
	Hyper‐CVAD	30			30.5	43.3	70	—	—	—	63.3^d^	6.7	
Almanza‐Huante [25] *Moderate ROB*	Modified CALGB C10403 (PIR)	27	Mar 2016–Jun 2019	1.8 years	24	44.4	85.2	55.6	21.4	> 30– > 100 × 10^3^/mL: 40.7	10.5^e^	—	Yes
	Modified ALL‐BFM 90 (PIR)	46		2.7 years	23.5	52.2	95.7	27.3	17.4	> 30– > 100 × 10^3^/mL: 28.9	42.9^e^	—	
	Hyper‐CVAD	137	Feb 2009–Jun 2015	8.4 years	20	46.7	95.6	48.9	6.6	> 30– > 100 × 10^3^/mL: 25	12.5^e^	—	
Buyukasik [33] *Moderate ROB*	CALGB‐8811 (PIR)	65	1999–2011	16.1 months	32	46.2	70.8	—	—	—	—	1.5	No
	Hyper‐CVAD with imatinib and RTX	57			29	52.6	64.9	—	—	—	—	7	
Demichelis [29] *Moderate ROB*	ALL‐BFM90 or CALGB 10403 (PIR)	73	Feb 2009–Jun 2015	BFM: 23.3 months CALGB: 12.7 months	24	49.3	91.8	—	—	> 30– > 100 × 10^3^/mcL: 33.3	31.9^f^	—	No
	Hyper‐CVAD	137		49.8 months	20	46.7	95.6	—	—	> 30– > 100 × 10^3^/mcL: 25.0	12.2^f^	—	
El‐Cheikh [42] *Moderate ROB*	Augmented BFM (PIR)	24	Nov 2000–Jan 2016	29 months	20	46	79	—	4.2	—	33^d^	—	No
	Hyper‐CVAD	38			38	50	89	—	47.4	—	76^d^	—	
Karcioglu [30] *Moderate ROB*	BFM95 (PIR)	23	2003–2016	56.5 months	23	21	78	—	—	—	—	17.4	No
	CALGB (PIR)	40		90.2 months	39	40	82	—	—	—	—	7.5	
	Hyper‐CVAD	37		22.6 months	35	48	75	—	—	—	—	18.9	
Lasheen [24] *High ROB*	BFM‐like (PIR)	51	2011–Jan 2019	—	28	36.4	62.3	—	—	—	—	—	Yes
	Hyper‐CVAD	26											
Li [23] *Moderate ROB*	Modified NHL‐BFM‐95 (PIR)	136	Jan 2000–Aug 2018	—	28	36	21.3	—	0	—	21.3^g^	2.2	Yes
	Hyper‐CVAD/MA	71			32	33.8	29.6	—	0	—	32.4^g^	2.8	
Peng [31] *Moderate ROB*	BFM90 (PIR)	60	Aug 2004–Oct 2007	42 months	40	—	68.3	—	—	> 30 × 10^6^/L: 30	—	—	No
	Hyper‐CVAD	35			42	—	65.7	—	—	> 30 × 10^6^/L: 37	—	—	
Rytting [26]^h^ *Moderate ROB*	Augmented BFM (PIR)	106	Oct 2006–Mar 2014	5.5 years	22	38.7	80.2	—	—	—	—	12.3	Yes
	Hyper‐CVAD	102	Nov 2002–July 2015	7.3 years	27	36.3	80.4	45.1	39.2	—	—	7.8	
Wann [34] *High ROB*	MASPORE (PIR)	29	Jan 2005–Jun 2021	4.5 years	23	—	—	—	—	—	—	—	No
	Hyper‐CVAD	87		10.8 years	28	—	—	—	—	—	—	—	

Abbreviations: ALL, acute lymphoblastic leukemia; ASP, asparaginase; BFM, Berlin‐Frankfurt‐Münster; B‐LBL, B‐cell lymphoblastic lymphoma; CALGB, Cancer and Leukemia Group B; CNS, central nervous system; DFCP, Dana Farber Consortium Protocol; hyper‐CVAD, hyper‐fractionated cyclophosphamide, vincristine, doxorubicin, and dexamethasone; MA, methotrexate and cytarabine; MASPORE, Malaysia‐Singapore Study Group; NCCN, National Comprehensive Cancer Network; NHL, Non‐Hodgkin's lymphoma; PIR, pediatric‐inspired regimen; ROB, risk of bias; RTX, rituximab; SLR, systematic literature review; WBC, white blood cell count.

^a^ Risk of bias for nonrandomized comparative studies was based on a Downs and Black score, and subjectively categorized into high risk of bias (score 0–13), moderate risk of bias (score 14–18), and low risk of bias (score 19–25).

^b^ All studies except Lasheen 2020, Karcioglu 2018, and Alabdulwahab 2017 reported median age. Lasheen 2020 and Karcioglu 2018 reported mean age. Alabdulwahab 2017 reported % patient < and ≥ 21 years.

^c^ High‐risk cytogenetics.

^d^ High risk.

^e^ High‐risk cytogenetics according to NCCN criteria.

^f^ High‐risk karyotype.

^g^ High risk according to the international prognostic index.

^h^ This study was excluded from the SLR because some patients in the hyper‐CVAD arm also received ASP.

Based on these retrospective, comparative studies, PIRs demonstrated improved or comparable results vs. hyper‐CVAD across all outcomes of interest (Table 2). Among response outcomes, four studies reported more favorable CR with PIRs than hyper‐CVAD. In two studies [29, 31], improvements in outcomes were statistically significant. In one study [25], outcomes were only significant at certain time points, whereas another study did not report the significance of the difference in CR compared with hyper‐CVAD [30]. Of five studies comparing relapse rate with PIRs vs. hyper‐CVAD, all reported equal or greater relapse rate in patients treated with hyper‐CVAD [25, 29, 32, 33, 34]. Duration of response was reported in one study and was found to be similar for patients treated with PIRs and hyper‐CVAD [35]. The percentage of patients achieving CR during follow‐up (no time point specified) ranged from 75% to 90.7% in the PIR group, and from 73.1% to 86.7% for hyper‐CVAD. One study reported on minimal residual disease (MRD) with MRD negativity in 69.1% of patients treated with PIRs and 60.1% of patients treated with hyper‐CVAD [23].

**TABLE 2:** Summary of study outcomes between PIRs and hyper‐CVAD.

Study	Survival		Response		Relapse/refractory (%)		Early/TR mortality (%)	
	PIR	Hyper‐CVAD	PIR	Hyper‐CVAD	PIR	Hyper‐CVAD	PIR	Hyper‐CVAD
Abbasi [35]	Median OS (95% CI) 30 mo (22, 47)	Median OS (95% CI) 30 mo (29, 43)	CR: 86**%** Median DoR (95% CI) 26 mo (21, 32)	CR: 90**%** Median DoR (95% CI) 28 mo (24, 35)	—	—	—	—
Alabdulwahab [32]	3‐year OS (±SE) 72.6 ± 6.6% 3‐year DFS (±SE) 70.9 ± 9.2%	3‐year OS (±SE) 48.5 ± 10.5% 3‐year DFS (±SE) 41.6 ± 11.4%%	CR: 90.7**%**	CR: 86.7**%**	Relapse: 23.3	Relapse: 36.7	—	—
Alacacioglu [36]	Mean OS 55.1 ± 4.9 mo RFS 53.9 ± 5.4 mo	Mean OS 41.5 ± 6.4 mo RFS 39.1 ± 6.8 mo	—	—	—	—	—	—
Almanza‐Huante [25]	**CALGB C10403:** Median OS (95% CI) 32.53 mo (14.02, 51.05) **ALL‐BFM 90:** Median OS (95% CI) 13.94 mo (9.72, 18.17) **Overall PIR:** Median OS (95% CI) 18.5 mo (13.61, 23.43)	Median OS (95% CI) 11.08 mo (7.33, 14.83)	**CALGB C10403:** 4‐week CR: 81.5**%** **ALL‐BFM 90:** 4‐week CR: 78.3**%** **Overall PIR:** 4‐week CR: 79.5**%**	4‐week CR: 64.2**%**	**CALGB C10403:** 33.3/3.7 **ALL‐BFM 90:** 51.2/19.6 **Overall PIR:** 44.1/13.7	60/18.9	**CALGB C10403:** Induction‐related mortality 3.7 **ALL‐BFM 90:** Induction‐related mortality 0 **Overall PIR:** Induction‐related mortality 1.4	Induction‐related mortality 8
Buyukasik [33]	Median OS (95% CI) 43.4 mo (26.8, 60.1) Median DFS (95% CI) 55.8 mo (31, 80.7)	Median OS (95% CI) 16.4 mo (8.3, 24.5) Median DFS (95% CI) 13.9 mo (4.7, 23.1)	CR: 73.8**%**	CR: 84.2**%**	Refractory disease: 6.1	Refractory disease: 10.5	20	5.2
Demichelis [29]	Median OS (95% CI) 19.0 mo (3.3, 25.4)	Median OS (95% CI) 11.1 mo (7.3, 14.8)	4‐week CR: 79.5**%**	4‐week CR: 64.2**%**	Relapse: 41.5	Relapse: 60.0	Induction‐related mortality 1.4	Induction‐related mortality 8.0
El‐Cheikh [42]	3‐year OS 76.9% 3‐year DFS 76.4%	3‐year OS 71.9% 3‐year DFS 54.7%	CR: 100**%**	CR: 89**%**	—	—	—	—
Karcioglu [30]	**BFM‐95:** 3‐year OS 89% 3‐year DFS 89% **CALGB:** 3‐year OS 53% 3‐year DFS 55%	3‐year OS 41% 3‐year DFS 40%	**BFM‐95:** CR: 100**%** **CALGB:** CR: 81**%**	CR: 70**%**	—	—	**BFM‐95:** 0**CALGB:** 38	11
Lasheen [24]	Median OS 28 mo	Median OS 12 mo	CR: 90.2**%**	CR: 73.1**%**	—	—	—	—
Li [23]	5‐year OS 53.9% 5‐year PFS 47.9%	5‐year OS 30.2% 5‐year PFS 25.9%	CR: 77.9**%**	CR: 66.2**%**	—	—	0	0
Peng [31]	42 mo OS 65% 42 mo EFS 60%	42 mo OS 46% 42 mo EFS 40%	CR: 93**%** [42 mo]	CR: 77**%** [42 mo]	—	—	—	—
Rytting [26]	5‐year OS 60%	5‐year OS 60%	CR: 93**%** 5‐year CRD: 53%	CR: 98**%** 5‐year CRD: 55%	Relapse: 37	Relapse: 38	72	77
Wann [34]	5‐year OS 4.4 years 5‐year PFS 4.2 years	5‐year OS 3.7 years 5‐year PFS 3.2 years	CR: 82.5**%**	CR: 80.5**%**	10.3	44.8	—	—

Abbreviations: ALL, acute lymphoblastic leukemia; BFM, Berlin‐Frankfurt‐Münster; CALGB, Cancer and Leukemia Group B; CI, confidence interval; CR, complete response; CRD, complete response duration; DFS, disease‐free survival; DoR, duration of response; EFS, event‐free survival; hyper‐CVAD, hyper‐fractionated cyclophosphamide, vincristine, doxorubicin, and dexamethasone; mo, months; OS, overall survival; PFS, progression‐free survival; PIR, pediatric‐inspired regimen; RFS, relapse‐free survival; SE, standard error; TR, treatment‐related.

In terms of survival, nine studies reported improved OS, two reported improved progression‐free survival (PFS), and six reported longer relapse‐free survival (RFS), EFS, or DFS with PIRs compared with hyper‐CVAD. ASP‐containing PIRs had statistically significant improvements in median OS (four studies) [24, 29, 34, 36], 2‐year OS (one study) [25], 3‐year OS (three studies) [30, 31, 32], and 5‐year OS (one study) [23] compared with hyper‐CVAD. Both studies reporting PFS outcomes reported a significantly better PFS with PIRs compared with hyper‐CVAD [23, 34]. Of the six studies reporting longer RFS, EFS, or DFS, three reported statistically significant differences [31, 33, 36].

Of 12 studies that compared PIRs with hyper‐CVAD, five reported adverse events (AEs) [23, 30, 32, 34, 36]. Among these, pancreatitis was the most frequently reported AE in patients treated with PIRs ranging from 0% to 11.6% in four studies. For patients treated with hyper‐CVAD, the incidence of pancreatitis was noted in four studies ranging from 0% to 8.5% of patients [23, 30, 32, 36]. Other commonly reported AEs include osteonecrosis and sepsis, with osteonecrosis more common in patients treated with PIRs (6.6%–11.6%) compared with patients treated with hyper‐CVAD (5.6%). However, one study noted osteonecrosis only in patients treated with PIRs [32]. One study reported sepsis in 2.3% and 10% of patients treated with a PIR and hyper‐CVAD, respectively [32].

### 3.2 Results of Meta‐Analysis

Fourteen studies that compared PIRs with hyper‐CVAD were evaluated for suitability of the meta‐analysis, including the 12 studies identified by the SLR and two additional studies (Figure 1). The additional studies were excluded from the SLR because further review of each study revealed patients in the hyper‐CVAD arm received treatment with some ASP. However, the inclusion criteria of the meta‐analysis only required no ASP during the induction or consolidation phases of patients in the hyper‐CVAD arm and patients in these studies received ASP during the maintenance and intensification phases. Three of these studies included ASP beyond consolidation [23, 25, 26].

Of these 14 studies, 12 were primary publications; only five met the criteria of ASP treatment during the induction or consolidation phase while excluding mixed Ph‐/Ph+ cases (Figure 1, Tables 1 and S8). One study [31] was carried out in a substantially older population than the rest with a median age range of 40–42 years versus mean/median of 20–32 years, and as such the noncomparable demographics resulted in the study being subsequently excluded.

Ultimately, four studies with comparable demographics were deemed suitable for meta‐analysis although patient characteristics were not reported consistently across studies (Table 1) [23, 24, 25, 26]. The median age (min, max) in each of the studies was generally similar (Almanza‐Huante et al.: modified CALGB 24 years [18, 41], modified ALL‐BFM‐90 23.5 years [18, 43], hyper‐CVAD 20 years [14, 39]; Lasheen et al. [mean, range]: 28 years [16, 60]; Li et al.: modified BFM‐95 28 years [not reported]; hyper‐CVAD 32 years [not reported]; Rytting et al.: augmented BFM 22 years [13, 39], hyper‐CVAD 27 years [15, 40]) [23, 24, 25, 26]. Three studies had a moderate risk of bias and one a high risk of bias. These studies all shared outcome measures of clinical relevance, including CR and OS. The PIRs used in these four studies were not identical, but the core components of the regimens were sufficiently similar to justify their pooling (Table S1). All four studies compared hyper‐CVAD to BFM or CALGB regimen variations in AYA populations (median/mean age 20–32 years).

A total of 702 patients from the four studies were included in the analysis, with 366 patients in the PIR arm and 336 patients in the hyper‐CVAD arm. Each study was weighted using the inverse‐variance method for the fixed‐ and random‐effects models; such that the study with the smallest standard error (SE) [23] had the largest weight (45.5%) in both models, whereas the study with the largest SE [26] was assigned the smallest weight (4.4%).

**FIGURE 2:**
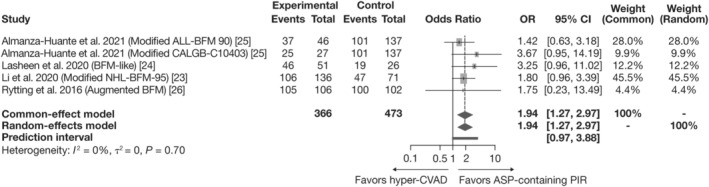
Meta‐analysis of PIR versus hyper‐CVAD for CR. ALL, acute lymphoblastic leukemia; ASP, asparaginase; BFM, Berlin‐Frankfurt‐Münster; CALGB, Cancer and Leukemia Group B; CI, confidence interval; CR, complete response; hyper‐CVAD, hyper‐fractionated cyclophosphamide, vincristine, doxorubicin, and dexamethasone; NHL, Non‐Hodgkin's lymphoma; OR, odds ratio; PIR, pediatric‐inspired regimen.

Patients treated with PIRs had a significantly higher chance of achieving CR than those treated with hyper‐CVAD. The pooled OR (95% CI) for the fixed‐ and random‐effects models were 1.94 (1.27, 2.97; Figure 2), indicating that patients treated with PIRs were approximately twice as likely to achieve CR compared with patients treated with hyper‐CVAD. The test of heterogeneity reported low variability across studies (*I* ^2^ = 0%, *p* = 0.70), which is supported by the identical results seen in the fixed‐ and random‐effects models. The PI (0.97, 3.88) marginally crosses the 1.00 boundary with most values above 1.

**FIGURE 3:**
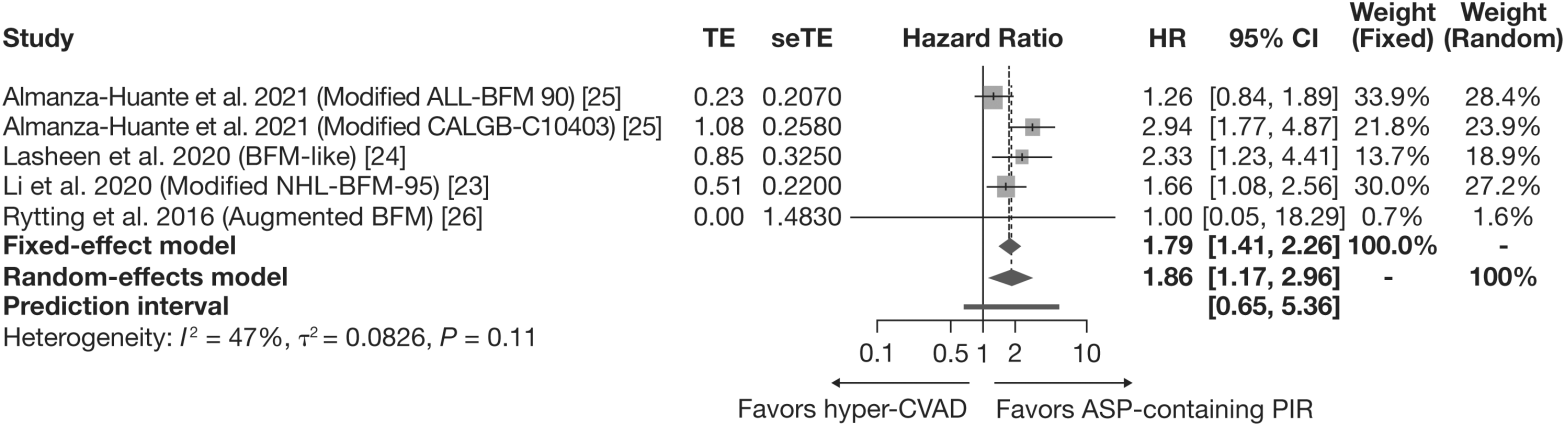
Meta‐analysis of PIR versus hyper‐CVAD for OS: Base case. ALL, acute lymphoblastic leukemia; ASP, asparaginase; BFM, Berlin‐Frankfurt‐Münster; CALGB, Cancer and Leukemia Group B; CI, confidence interval; HR, hazard ratio; hyper‐CVAD, hyper‐fractionated cyclophosphamide, vincristine, doxorubicin, and dexamethasone; NHL, Non‐Hodgkin's lymphoma; OS, overall survival; PIR, pediatric‐inspired regimen; seTE, standard error of the treatment effect; TE, treatment effect natural log (HR).

For the meta‐analysis of OS, two analyses—a base case and a scenario analysis—were considered. In the base case, patients treated with PIRs had a significantly lower risk of mortality compared with those treated with hyper‐CVAD (Figure 3). The pooled HR (95% CI) for the fixed‐effects and random‐effects models were 1.79 (1.41, 2.26) and 1.86 (1.17, 2.96), respectively. This indicates that patients treated with PIRs were approximately 1.8 times more likely to survive than patients treated with hyper‐CVAD. The heterogeneity analysis of these results revealed a moderate level of variability among the included studies (*I* ^2^ = 47%, *p* = 0.11). As with the previous analysis, the PI marginally crosses the 1.00 boundary, but most of the interval is above 1.

**FIGURE 4:**
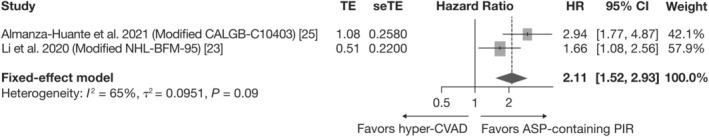
Meta‐analysis of PIR versus hyper‐CVAD of OS: Scenario analysis. ASP, asparaginase; BFM, Berlin‐Frankfurt‐Münster; CALGB, Cancer and Leukemia Group B; CI, confidence interval; HR, hazard ratio; hyper‐CVAD, hyper‐fractionated cyclophosphamide, vincristine, doxorubicin, and dexamethasone; NHL, Non‐Hodgkin lymphoma; OS, overall survival; PIR, pediatric‐inspired regimen; seTE, standard error of the treatment effect; TE, treatment effect natural log (HR).

An additional scenario analysis only included studies for which it was believed the assumption of proportionality of the HR was held, which assessed the impact of studies that may provide a source of bias [23, 25]. The random‐effects model was not considered in this scenario since this approach was unlikely to provide meaningful results given the limited number of studies included in this analysis. The meta‐analysis for OS in this scenario indicates substantial variability among the included studies (*I* ^2^ = 65%, *p* = 0.09) but revealed similar results for OS. Based on the pooled HR for the fixed‐effects model (2.11 [95% CI: 1.52, 2.93], Figure 4), patients receiving PIRs had a significantly lower risk of mortality compared with those treated with hyper‐CVAD.

## 4 Discussion

We present the results of the first, to our knowledge, SLR and meta‐analysis comparing PIRs to hyper‐CVAD for the treatment of ALL/LBL, which suggest AYA patients treated with pediatric‐inspired, ASP‐containing regimens have better outcomes than those treated with hyper‐CVAD. The SLR identified studies within which patients treated with PIRs had longer OS, PFS, and RFS/EFS/DFS than those treated with hyper‐CVAD. Additionally, the meta‐analysis suggested that PIRs are associated with improved rates of CR and OS in AYA patients with Ph‐ALL/LBL. Both the base case and scenario analysis indicated moderate and substantial variability, respectively, among the included studies but revealed similar results with regard to OS.

These findings align with previous reviews of PIRs in ALL/LBL in AYA populations [6, 9]. Of the 11 studies included in a 2012 meta‐analysis (none of which were RCTs), AYA patients treated with PIRs had a significant reduction in all‐cause mortality and increase in CR compared with conventional adult chemotherapy regimens, although none of these were hyper‐CVAD [6].

Although PIRs have been tested in adult populations, toxicities increase and survival decreases in adults ≥ 55 years [37]. Intense chemotherapy involving ASP is poorly tolerated in older patients leading to increased treatment‐related mortality. Thus, researchers propose using modified PIRs in older patients to manage treatment‐related toxicities [12, 37]. In this review, although noting it is not a comprehensive review of safety as not all comparative studies reported safety data, PIRs were associated with a slightly increased risk of pancreatitis and osteonecrosis. Despite greater toxicities from ASP‐containing PIRs in AYA compared with younger patients, the benefit‐to‐toxicity ratio of these regimens is favorable [9].

Results from this meta‐analysis suggesting favorable outcomes with ASP‐containing PIRs are consistent with current guidelines that recommend PIRs for AYA patients with ALL/LBL [5, 6, 8, 9, 38]. However, despite clinical guidelines, treatment varies substantially for this population, depending on whether they receive care in pediatric or adult cancer settings. A 2018 population‐based study [38] reporting on treatment patterns in AYA patients diagnosed with ALL between 2004 and 2014 showed 35% and 68% of AYA patients were treated in pediatric and adult cancer settings, respectively. Although this study identified survival advantages associated with PIRs, only ~25% of AYA patients in this study treated in adult cancer settings received PIRs.

Similar results have been recently reported in a 2023 retrospective study that describes the patterns of care in AYAs with newly diagnosed ALL [39]. Of 378 patients, 230 (61%) were treated with a PIR and 148 (39%) were treated with an adult regimen, most of which were hyper‐CVAD‐based (*n* = 125, 85%). Patients were more likely to be treated with adult regimens if they were older (20–39 years), male, and obese. Adult regimens were also more commonly used in small to medium‐sized hospitals, for treatment of patients on public insurance, and for those treated by nonpediatric oncologists. Treatment for AYA patients largely depends on referral patterns and hospital guidelines, but can sometimes also be due to chance, and further research is needed to fully understand the rationale behind these patterns [38, 39].

This analysis has limitations. Only four studies met the inclusion criteria for this meta‐analysis of PIRs versus hyper‐CVAD. While our results present low to substantial variability with CIs that did not cross the line of no difference (i.e., OR/HR = 1), a larger sample size is desirable. Furthermore, in the meta‐analysis for CR and OS, the PIs were wide and included values ≤ 1, suggesting that the results may not be representative of the entire population with ALL/LBL. It is important to note that PIs explore a different element of uncertainty than CIs; CIs measure the sampling error within the meta‐analytical model, which allows an estimated range for the true value of the treatment effect, whereas PIs estimate the results of a theoretical future study. PIs are subject to greater uncertainty than CIs, thus, their range will always be wider than that of CIs. Knowing that most of the PI ranges were values > 1, we may reasonably presume that the chance of discordant results occurring in the future is relatively small.

Further limitations included study design and patient population of the included studies. All included studies that compared PIRs with hyper‐CVAD were nonrandomized comparison studies with at least moderate risk of bias; therefore, the results would not be as robust as those from RCTs. The type of ASP used in the PIRs varied (one study assessed L‐asparaginase regimens, two assessed pegaspargase, and one did not specify), and the details of patients' clinical characteristics were reported inconsistently, limiting comparability of the patient populations. Additionally, while conventional hyper‐CVAD regimens are relatively homogenous, PIRs are more heterogenous, despite the traditional BFM backbone, due to the combination of administered agents and varying blocks of therapy; this represents a barrier in analyzing them as a uniform comparison group. For example, five of the studies in the SLR confirmed that ASP was administered beyond the consolidation phase in the PIRs. While the role of ASP administered after the induction and consolidation phases of therapy is outside the scope of this SLR, this approach may contribute to improved survival outcomes and CR rates with PIRs [23, 25, 26]. The potential heterogeneity in cultural, genetic, and healthcare practices in the different countries where the studies took place (i.e., China, Egypt, Jordan, Lebanon, Mexico, Saudi Arabia, Singapore, Turkey, and the United States) could contribute to differences in reported outcomes. The importance of ethnicity in ALL has been studied and may play a role in disease incidence/tumor genetics as well as response to treatment [29, 40]; however, we believe the variability of the examined populations could also be considered advantageous when interpreting outcomes. Thus, without head‐to‐head studies, there was no way to address whether treatment cost or institutional bias affected the results.

Furthermore, recent advances in ALL treatment such as the incorporation of targeted agents inotuzumab or blinatumomab were not captured in this analysis [41]. Two studies included in the SLR involved rituximab, but were not part of the meta‐analysis [33, 42], so the impact of rituximab on treatment outcomes has not been determined. These agents are being incorporated into both standard hyper‐CVAD and PIR chemotherapy backbones. Notably, the addition of blinatumomab to consolidation chemotherapy in a pediatric‐inspired protocol significantly improved OS and DFS in patients with B‐lymphoblastic leukemia in MRD‐negative remission [43, 44, 45]. Future studies should evaluate the relative efficacy and safety/tolerability of the modified hyper‐CVAD and PIR with the addition of these targeted agents.

A strength of the meta‐analysis is that half of the studies compared treatment arms within the same centers, which has not been previously explored in prior analyses. Future prospective head‐to‐head studies are required to confirm these results.

## 5 Conclusion

The results of this meta‐analysis suggest that AYAs with ALL/LBL treated with ASP‐containing PIRs could have better clinical outcomes than those treated with hyper‐CVAD. Although ASP‐containing PIRs in AYAs are associated with greater toxicities compared with younger patients, the benefit‐to‐toxicity ratio of these regimens is still favorable. The paucity of prospective RCTs illustrates the need for future studies comparing these regimens.

## 6 Summary Points

- Adolescents and young adults (AYAs) with acute lymphoblastic leukemia/lymphoblastic lymphoma (ALL/LBL) have poorer outcomes than pediatric patients.
- Retrospective studies have suggested that treatment of AYAs with ALL/LBL with pediatric‐inspired regimens (PIRs) can improve response rate and survival outcomes compared with traditional adult regimens.
- This systematic literature review (SLR) and meta‐analysis compared asparaginase (ASP)‐containing PIRs with hyper‐fractionated cyclophosphamide, vincristine, doxorubicin, and dexamethasone (hyper‐CVAD) in the treatment of ALL/LBL in AYA patients.
- The studies identified in the SLR (median age 20–42 years) reported improved longer overall survival and progression free survival, as well as longer relapse‐free/event‐free/disease‐free survival in patients receiving ASP‐containing PIRs compared with those receiving hyper‐CVAD.
- The meta‐analysis found that complete response was approximately twice as likely in patients receiving ASP‐containing PIRs than those receiving hyper‐CVAD, and survival was 1.8 times more likely.
- Although noting greater toxicities associated with ASP‐containing PIRs in AYAs compared with younger patients, the benefit‐to‐toxicity ratio of these regimens is still favorable.
- Overall, the results of this study suggest that AYA patients treated with pediatric‐inspired, ASP‐containing regimens have better outcomes than those treated with hyper‐CVAD.

## Ethics Statement

The authors have nothing to report.

## Consent

The authors have nothing to report.

## Conflicts of Interest

W.S. and M.S. are employees of Jazz Pharmaceuticals and hold stock or stock options in Jazz Pharmaceuticals. A.M. is an employee of Crystallize, which was contracted by Jazz Pharmaceuticals to conduct the systematic literature review. J.B. is the founder of JB Medical, which was contracted by Jazz Pharmaceuticals to conduct the meta‐analyses. E.K. was a former employee of JB Medical, which was contracted by Jazz Pharmaceuticals to conduct the meta‐analyses. D.J.D. has consulted for Agios, Amgen, Autolus, Blueprint Pharmaceuticals, Forty‐Seven, Gilead, Incyte, Jazz Pharmaceuticals, Novartis, Pfizer, Servier Pharmaceuticals, and Takeda, and has received research support from AbbVie, Blueprint Pharmaceuticals, GlycoMimetics, and Novartis.

## Supporting information


**Data S1.** Supporting Information.

## Data Availability

All data underlying the results are available as part of the article and no additional source data are required.
