# Supplementary material for: The Efficacy of Pediatric‐Inspired Regimens vs. Hyper‐CVAD in the Treatment of Adolescents and Young Adults With Acute Lymphoblastic Leukemia: A Systematic Review and Meta‐Analysis
Source: Am J Hematol. 2025 Feb 13;100(5):847–59. doi: 10.1002/ajh.27607 (PMC11966355; doi:10.1002/ajh.27607)
Supplement: Supplementary file 1 — Data S1. Supporting Information. [file AJH-100-847-s001.docx]

## SUPPLEMENTARY MATERIALS

**Table S1. Components of treatment regimens in PIR arms of the included* studies.**

| **Almanza-Huante 2021^25^** | Modified ALL-BFM 90 | **Induction** Prednisone  Daunorubicin  Vincristine  L-asparaginase | **Consolidation** **1**  IV MTX  IV ARA-C  Mercaptopurine  Vincristine  Folinic acid  Dexamethasone  L-asparaginase | **Consolidation** **2**  IV MTX  Mercaptopurine  Vincristine  Folinic acid  Dexamethasone  Ifosfamide Daunorubicin  L-asparaginase  IT MTX/ARA-C | **Consolidation** **3**  Dexamethasone  IV ARA-C  Etoposide  L-asparaginase  IT MTX/ARA-C | | **Maintenance**  Mercaptopurine  MTX  Prednisone  Vincristine  L-asparaginase |
| --- | --- | --- | --- | --- | --- | --- | --- |
|  | Modified CALGB-C10403 | **Induction** Dexamethasone  Daunorubicin  Vincristine  L-asparaginase | **Consolidation 1**  Cyclophosphamide  IV ARA-C  Mercaptopurine  Vincristine  L-asparaginase | **Consolidation 2**  IV MTX  Vincristine  Folinic acid  L-asparaginase  IT MTX/ARA-C | **Delayed intensification**  Dexamethasone  Vincristine  Doxorubicin  IV ARA-C  Cyclophosphamide  Mercaptopurine  L-asparaginase | | **Maintenance**  Mercaptopurine  MTX  Dexamethasone  Vincristine |
| **Lasheen 2020^24^** | BFM-like | Detailed regimen not given in reference (abstract only) | | | | | |
| **Li 2020^23^** | Modified NHL BFM-95 | **Induction 1A**  Prednisone  Pirarubicin  Vincristine  Pegaspargase  IT MTX/ARA-C | **Induction 1B**  Cyclophosphamide  IV ARA-C  Mercaptopurine  IT MTX/ARA-C | **Protocol M**  Mercaptopurine  MTX  IT MTX | **Reinduction IIA**  Dexamethasone  Pirarubicin  Vincristine  Pegaspargase | **Reinduction IIB**  Cyclophosphamide  IV ARA-C  Thioguanine  IT MTX/ARA-C | **Maintenance**  MTX  Mercaptopurine  IV MTX |
| **Rytting 2016^26^** | Augmented BFM | **Induction** Prednisone  Daunorubicin  Vincristine  Pegaspargase  IT ARA-C  IT MTX | **Consolidation 1**  Cyclophosphamide  IV ARA-C  Mercaptopurine  Vincristine  Pegaspargase  IT MTX | **Consolidation 2**  IV MTX  Vincristine  Pegaspargase  IT MTX | **Consolidation 3A**  Doxorubicin  Dexamethasone  Vincristine  Pegaspargase  IT MTX | **Consolidation 3B**  Cyclophosphamide  IV ARA-C  Thioguanine  Vincristine  Pegaspargase  IT MTX | **Maintenance**  Mercaptopurine  MTX  Dexamethasone  Vincristine  IT MTX |
| **Abbasi 2013^35,a^** | CALGB 8811 | **Induction**  Cyclosporine  Daunorubicin  Vincristine  Prednisone  L-asparaginase | **Early intensification**  Cyclosporine  MTX  6-mercaptopurine  ARA-C  Vincristine  L-asparaginase | **Interim maintenance**  IT MTX  6-Mercaptopurine  MTX | **Late intensification**  Doxorubicin  Vincristine  Dexamethasone  Cyclophosphamide  6-Thioguanine  ARA-C | **Prolonged maintenance**  Vincristine  Prednisone  MTX  6-Mercaptopurine |  |
| **Alabdulwahab 2017^32,b^** | Modified DFCP | **Induction**  Prednisone  Doxorubicin  Vincristine  MTX  L-asparaginase | **CNS therapy**  Doxorubicin  Vincristine  6-Mercaptopurine  IT MTX/ARA-C/hydrocortisone | **Intensification**  Doxorubicin  Vincristine  6-mercaptopurine  Dexamethasone  L-asparaginase  IT MTX  IT MTX/ARA-C/hydrocortisone | **Maintenance**  Vincristine  6-Mercaptopurine  Dexamethasone  MTX  IT-MTX/ARA-C/hydrocortisone |  |  |
| **Alacacioglu 2014^36^** | BFM | **Induction**  Vincristine  Daunorubicin/ doxorubicin  Prednisone  L-asparaginase | **Early consolidation**  Cyclophosphamide  ARA-C  6-Mercaptopurine  IT MTX | **Interim maintenance**  6-Mercaptopurine  MTX | **Delayed intensification: Reinduction**  Dexamethasone  Vincristine  Daunorubicin/doxorubicin | **Delayed intensification : Reconsolidation**  Cyclophosphamide  ARA-C  6-Thioguanine |  |
| **Buyukasik 2013^33^** | CALGB-8811 | **Induction**  Cyclophosphamide  Daunorubicin  Vincristine  Prednisone  L-asparaginase | **Early intensification**  Cyclophosphamide  6-Mercaptopurine  ARA-C  Vincristine  L-asparaginase  IT MTX | **Interim maintenance**  IT-MTX  6-Mercaptopurine  MTX | **Late intensification**  Doxorubicin  Vincristine  Dexamethasone  Cyclophosphamide  6-Thioguanine  ARA-C | **Maintenance**  6-mercaptopurine  Vincristine  MTX  Prednisone |  |
| **Demichelis 2019^29^** | ALL-BFM90 | Detailed regimen not given in reference (abstract only) | | | | | |
|  | CALGB 10403 | Detailed regimen not given in reference (abstract only) | | | | | |
| **El-Cheikh 2017^40^** | Augmented BFM | **Induction** Prednisone  Daunorubicin  Vincristine  Pegaspargase  IT ARA-C  IT MTX | **Consolidation 1**  Cyclophosphamide  IV ARA-C  Mercaptopurine  Vincristine  Pegaspargase  IT MTX | **Consolidation 2**  IV MTX  Vincristine  Pegaspargase  IT MTX | **Consolidation 3A**  Doxorubicin  Dexamethasone  Vincristine  Pegaspargase  IT MTX | **Consolidation 3B**  Cyclophosphamide  IV ARA-C  Thioguanine  Vincristine  Pegaspargase  IT MTX | **Maintenance**  Mercaptopurine  MTX  Dexamethasone  Vincristine  IT MTX |
| **Karcioglu 2018^30^** | BFM95 | Detailed regimen not given: Induction, consolidation, re-induction, and maintenance phases involve prednisolone, vincristine, daunorubicin, and L-asparaginase | | | | | |
|  | CALGB | Detailed regimen not given: regimen involves cyclophosphamide, prednisolone, vincristine, daunorubicin, and L-asparaginase | | | | | |
| **Peng 2020^31^** | BFM90 | Detailed regimen not given | | | | | |
| **Wann 2021^34^** | MASPORE | Detailed regimen not given (abstract only): regimen involves L-asparaginase, vincristine, dexamethasone (intermediate risk) or L-asparaginase, vincristine, dexamethasone, daunorubicin (high risk) | | | | | |

Abbreviations: ALL, acute lymphoblastic leukemia; ARA-C, cytarabine; BFM, Berlin-Frankfurt-Münster; CALGB, Cancer and Leukemia Group B; CNS, central nervous system; DFCP, Dana Farber Consortium Protocol; IT, intrathecal; IV, intravenous; MASPORE, Malaysia-Singapore Study Group; MTX, methotrexate PIR, pediatric-inspired regimen.

*Included studies comprise 12 studies included in both the SLR and meta-analysis and 1 study included in the meta-analysis only

^a^Regimen described in Larsen 1995^41^.

^b^Regimen described in Storring 2009^42^.

Note: The red font in the table signifies asparaginase in any of the treatment cycles.

### Appendix A: Search Strategy

**Table S2. Embase search strategy.** Embase was searched on April 19, 2022.

|  | Search terms | Number of hits |
| --- | --- | --- |
| 1 | ('acute lymphoblastic leukemia'/exp OR 'acute lymphoblastic leukemia' OR all:ab,ti OR lbl:ab,ti OR (acute NEAR/2 leukemia) OR 'lymphoblastoma'/exp OR 'lymphoblastoma' OR 'lymphoblastic lymphoma':ab,ti) AND [humans]/lim AND [abstracts]/lim | 5,346,190 |
| 2 | ('asparaginase'/exp OR 'asparaginase' OR 'asparaginase macrogol'/exp OR 'asparaginase macrogol' OR L-asparaginase:ab,ti OR pegaspargase:ab,ti OR erwinase:ab,ti OR erwinaze:ab,ti OR crisantaspase:ab,ti OR BFM:ab,ti OR GBTLI-ALL-99:ab,ti OR CALGB:ab,ti OR AALL:ab,ti OR P9407:ab,ti OR china-98:ab,ti OR CCG:ab,ti OR DFCI:ab,ti OR DFCP:ab,ti OR ‘ECOG E2993’:ab,ti OR graspa:ab,ti OR euro-lb02:ab,ti OR fralle-93:ab,ti OR goleal1:ab,ti OR interfant-99:ab,ti OR lalpof:ab,ti OR lmt81:ab,ti OR msk-ny-ii:ab,ti OR nhl13:ab,ti OR nopho:ab,ti OR sjcrh:ab,ti OR ‘total therapy xv’:ab,ti OR tpog:ab,ti OR ukall:ab,ti OR ‘usa mcp-841’:ab,ti OR hyper-cvad:ab,ti OR (cyclophosphamide AND doxorubicin AND vincristine AND methotrexate AND dexamethasone AND cytarabine)) AND [humans]/lim AND [abstracts]/lim | 20,147 |
| 3 | 1 AND 2 | 13,968 |
| 4 | (response*:ab,ti OR respond*:ab,ti OR surviv*:ab,ti OR OS:ab,ti OR PFS:ab,ti OR EFS:ab,ti OR DFS:ab,ti OR RFS:ab,ti OR death*:ab,ti OR died:ab,ti OR mortality:ab,ti OR 'minim* residual disease':ab,ti OR MRD:ab,ti OR relaps*:ab,ti OR refractory:ab,ti OR complet*:ab,ti OR safe*:ab,ti OR toxic*:ab,ti OR ((adverse OR side) NEAR/1 (effect* OR event*))) AND [humans]/lim AND [abstracts]/lim | 7,723,547 |
| 5 | 3 AND 4 | 11,634 |

**Table S3. PubMed search strategy.** PubMed was searched on April 21, 2022.

|  | Search terms | Number of hits |
| --- | --- | --- |
| 1 | all[Title/Abstract] OR lbl[Title/Abstract] OR "Leukemia, Lymphoid"[Mesh] OR "Precursor Cell Lymphoblastic Leukemia-Lymphoma"[Mesh] OR "acute lymphoblastic leukemia" OR "acute lymphocytic leukaemia" OR "acute lymphoblastic leukemia" OR "acute lymphoblastic leukaemia" OR lymphoblastoma OR "lymphoblastic lymphoma" Filters: Abstract, Humans | 64,168 |
| 2 | asparaginase[Mesh] OR "asparaginase macrogol"[Mesh] OR pegaspargase[Title/Abstract] OR L-asparaginase[Title/Abstract] OR erwinase[Title/Abstract] OR erwinaze[Title/Abstract] OR crisantaspase[Title/Abstract] OR BFM[Title/Abstract] OR GBTLI-ALL-99[Title/Abstract] OR CALGB[Title/Abstract] OR AALL[Title/Abstract] OR P9407[Title/Abstract] OR china-98[Title/Abstract] OR CCG[Title/Abstract] OR DFCI[Title/Abstract] OR DFCP[Title/Abstract] OR "ECOG E2993"[Title/Abstract] OR graspa[Title/Abstract] OR euro-lb02[Title/Abstract] OR fralle-93[Title/Abstract] OR goleal1[Title/Abstract] OR interfant-99[Title/Abstract] OR lalpof[Title/Abstract] OR lmt81[Title/Abstract] OR msk-ny-ii[Title/Abstract] OR nhl13[Title/Abstract] OR nopho[Title/Abstract] OR sjcrh[Title/Abstract] OR "total therapy xv"[Title/Abstract] OR tpog[Title/Abstract] OR ukall[Title/Abstract] OR "usa mcp-841"[Title/Abstract] OR hyper-cvad[Title/Abstract] OR (cyclophosphamide AND doxorubicin AND vincristine AND methotrexate AND dexamethasone AND cytarabine)[Title/Abstract] Filters: Abstract, Humans | 6,497 |
| 3 | 1 AND 2 | 2,952 |
| 4 | response*[Title/Abstract] OR respond*[Title/Abstract] OR surviv*[Title/Abstract] OR OS[Title/Abstract] OR PFS[Title/Abstract] OR EFS[Title/Abstract] OR DFS[Title/Abstract] OR RFS[Title/Abstract] OR death*[Title/Abstract] OR died[Title/Abstract] OR mortality[Title/Abstract] OR "minimal residual disease" "minimum residual disease"[Title/Abstract] OR MRD[Title/Abstract] OR relaps*[Title/Abstract] OR refractory[Title/Abstract] OR complet*[Title/Abstract] OR safe*[Title/Abstract] OR toxic*[Title/Abstract] OR ((adverse OR side) AND (effect* OR event*)) Filters: Abstract, Humans | 3,258,259 |
| 5 | 3 AND 4 | 2,305 |

**Table S4. Cochrane library search strategy.** The Cochrane library was searched on April 19, 2022.

|  | Search terms | Number of hits |
| --- | --- | --- |
| 1 | MeSH descriptor [Leukemia, Lymphoid] explode all trees OR ‘acute lymphocytic leukemia’:ti,ab OR ‘acute lymphocytic leukaemia’:ti,ab OR ‘acute lymphoblastic leukemia’:ti,ab OR ‘acute lymphoblastic leukaemia’:ti,ab OR lymphoblastoma:ab,ti OR 'lymphoblastic lymphoma':ab,ti | 4,076 |
| 2 | asparaginase' OR 'asparaginase macrogol' OR L-asparaginase:ab,ti OR pegaspargase:ab,ti OR erwinase:ab,ti OR erwinaze:ab,ti OR crisantaspase:ab,ti OR BFM:ab,ti OR GBTLI-ALL-99:ab,ti OR CALGB:ab,ti OR AALL:ab,ti OR P9407:ab,ti OR china-98:ab,ti OR CCG:ab,ti OR DFCI:ab,ti OR DFCP:ab,ti OR ‘ECOG E2993’:ab,ti OR graspa:ab,ti OR euro-lb02:ab,ti OR fralle-93:ab,ti OR goleal1:ab,ti OR interfant-99:ab,ti OR lalpof:ab,ti OR lmt81:ab,ti OR msk-ny-ii:ab,ti OR nhl13:ab,ti OR nopho:ab,ti OR sjcrh:ab,ti OR ‘total therapy xv’:ab,ti OR tpog:ab,ti OR ukall:ab,ti OR ‘usa mcp-841’:ab,ti OR hyper-cvad:ab,ti OR (cyclophosphamide AND doxorubicin AND vincristine AND methotrexate AND dexamethasone AND cytarabine) | 2,352 |
| 3 | 1 AND 2 | 992 |

In total, 12,165 abstracts were screened from databases after deduplication.

**Gray Literature Search Strategy**

The gray literature conference sites listed below were searched to identify additional relevant abstracts from 2020–2022. Individual searches were carried out in turn for the following key words: “acute lymphoblastic leukaemia”, “acute lymphoblastic leukemia”, “lymphoblastic lymphoma”, “LBL”, “ALL”. Any relevant abstracts were added to the list of abstracts for screening.

**Table S5. Gray literature search strategy.**

| Source | Number of relevant hits |
| --- | --- |
| American Society of Clinical Oncology (ASCO) | 0 |
| European Society for Medical Oncology (ESMO) | 0 |
| International Society for Pharmacoeconomics and Outcomes Research (ISPOR) | 0 |
| American Society of Hematology (ASH) | 9 |
| European Haematology Association (EHA) | 3 |
| American Association for Cancer Research (AACR) | 0 |
| National Comprehensive Cancer Network (NCCN) guidelines | 0 |
| European Society for Medical Oncology (ESMO) guidelines | 2 |
| Clinicaltrials.gov | 0 |
| European Union Clinical Trials Register | 0 |

An additional 14 abstracts were identified for screening.

The reference lists of relevant SLRs were screened for relevant citations. Three new citations were assessed at full text screening but all were excluded.

**Table S6. Inclusion and exclusion criteria.**

| **Criterion** | **Inclusion criterion** | **Exclusion criterion** |
| --- | --- | --- |
| Disease | ALL  LBL  Newly diagnosed or relapsed/refractory | Other types of cancer  Burkitt’s lymphoma  Hodgkin’s lymphoma |
| Population | Adults  Adolescent/young adult  Children | Animals  Laboratory samples or cell cultures |
| Interventions | Any line of chemotherapy with regimen containing one of the following:  L-asparaginase  Pegaspargase  Erwinia-derived asparaginase  Calaspargase  Any other asparaginase  Hyper-CVAD (any variant of this regimen, including with asparaginase) | Other chemotherapy regimens with no asparaginase at any stage  Studies assessing outcomes in mixed populations (patients with a mixture of malignancies, not just ALL/LBL) or those receiving different chemotherapy regimens (not just asparaginase- or hyper-CVAD-based regimens) where outcomes are unlikely to be reported by regimen |
| Comparators | Any specific chemotherapy regimen  Standard of care  No active treatment | Comparison groups who did not all receive the same treatment regimen  Non-chemotherapy interventions such as Stem cell transplant |
| Outcomes | Response rates  Duration of response  Survival rates: overall, disease-free, progression-free, event-free, relapse-free  Completion of scheduled chemotherapy regimen  Adverse events including hypersensitivity, myelosuppression, hepatotoxicity, thrombosis, pancreatitis  Relapse rates  Time to relapse  Refractory disease rates  MRD rates | Pharmacokinetics/pharmacodynamics of asparaginase  Survival or response rates only described as function of MRD status  Analyses only exploring factors or mutations associated with response or survival  Management of adverse events of chemotherapy |
| Study methodology | RCTs  Single-arm clinical trials  Retrospective or prospective observational studies  Cross-sectional or case-control studies  Systematic reviews of relevant studies | Editorials, letters, opinion-piece articles  Conference abstracts with a corresponding full-text paper and not reporting new data  Study protocols with no results |
| Study size | RCTs; any  Single-arm clinical trials and observational studies: 20 or more participants in each treatment group treated with asparaginase or hyper-CVAD | Single-arm clinical trials and observational studies: <20 participants receiving asparaginase |
| Language | Any |  |
| Publication date | Conference abstracts: 2019 onwards  Full text publications: any | Conference abstracts published before 2019 that do not have useful data |

Abbreviations: ALL, acute lymphoblastic leukemia; hyper-CVAD, hyper-fractionated cyclophosphamide, vincristine, doxorubicin, and dexamethasone; LBL, lymphoblastic lymphoma; MRD, minimal residual disease; RCT, randomized controlled trial.

### Appendix B: Studies excluded at full-text screening

**Table S7: SLR excluded studies at full-text screening.**

| Study | Reason for exclude |
| --- | --- |
| Advani, S. H., et al. (1989). "Acute lymphoblastic leukemia in childhood: treatment, results and prognostic factors." Indian J Cancer 26(3): 180-8. | Full text irretrievable |
| Albertsen, B.K., et al. (2019). "Intermittent Versus Continuous PEG-Asparaginase to Reduce Asparaginase-Associated Toxicities: a NOPHO ALL2008 Randomized Study." Journal of Clinical Oncology 37(19): 1638-1646. | ASP vs. ASP comparison |
| Al-Khabori, M., et al. (2010). "Improved survival using an intensive, pediatric-based chemotherapy regimen in adults with T-cell acute lymphoblastic leukemia." Leuk Lymphoma 51(1): 61-5. | Irrelevant comparator |
| Al-Nasser, A., et al. (2008). "Improved outcome for children with acute lymphoblastic leukemia after risk-adjusted intensive therapy: A single-institution experience." Annals of Saudi Medicine 28(4): 251-259. | ASP vs. ASP comparison |
| Anderson, J., et al. (1981). "Comparison of the therapeutic response of patients with childhood acute lymphoblastic leukemia in relapse to vindesine versus vincristine in combination with prednisone and L-asparaginase: a phase III trial." Cancer Treatment Reports 65(11-12): 1015-1019. | ASP vs. ASP comparison |
| Annonymous., (2021). "L-ASPARAGINASE HYPERSENSITIVITY AND SURVIVAL RELATIONSHIP IN TURKISH PEDIATRIC ACUTE LYMPHOBLASTIC LEUKEMIA PATIENTS TREATED WITH MODIFIED ST. JUDE TOTAL XV TREATMENT PROTOCOL." EHA 2021. | Irrelevant comparator |
| Apostolidou, E., et al. (2020). "ALL-249: Clinical Outcomes of Patients with Newly Diagnosed Acute Lymphoblastic Leukemia in a County Hospital System." Clinical Lymphoma, Myeloma and Leukemia 20: S166-S167. | No relevant outcomes |
| Attarbaschi, A., et al. (2020). "Randomized post-induction and delayed intensification therapy in high-risk pediatric acute lymphoblastic leukemia: long-term results of the international AIEOP-BFM ALL 2000 trial." Leukemia 34(6): 1694-1700. | ASP vs. ASP comparison |
| Aur, R.J., et al. (1978). "Childhood acute lymphocytic leukemia: study VIII." Cancer 42(5): 2123-2134. | Irrelevant comparator |
| Avramis, V.I., et al. (2002). "A randomized comparison of native Escherichia coli asparaginase and polyethylene glycol conjugated asparaginase for treatment of children with newly diagnosed standard-risk acute lymphoblastic leukemia: a Children's Cancer Group study." Blood 99(6): 1986-1994. | ASP vs. ASP comparison |
| Badowska, W. (2008). "Analysis of therapy results and prognostic factors in children with acute lymphoblastic leukaemia in Warmia and Mazury region: 17-years experience." Medycyna wieku rozwojowego 12(4 Pt 2): 1001-1007. | ASP vs. ASP comparison |
| Borbolla-Escoboza, J. R., et al. (1998). "Comparison of 2 regimens of polychemotherapy in refractory acute leukemia or in relapse." Revista de investigación clínica; organo del Hospital de Enfermedades de la Nutrición 50(4): 307-310. | Irrelevant comparator |
| Brandalise, S.R., et al. (2010). "Benefits of the intermittent use of 6-mercaptopurine and methotrexate in maintenance treatment for low-risk acute lymphoblastic leukemia in children: Randomized trial from the Brazilian childhood cooperative group - Protocol ALL-99." Journal of Clinical Oncology 28(11): 1911-1918. | Irrelevant comparator |
| Brigitha, L. J., et al. (2022). "Hypersensitivity to Pegylated E.colia sparaginase as first-line treatment in contemporary paediatric acute lymphoblastic leukaemia protocols: a meta-analysis of the Ponte di Legno Toxicity working group." European Journal of Cancer 162: 65-75. | Systematic literature review |
| Brown, P., et al. (2020). "Ukallr3 reinduction is toxic and ineffective for early first bone marrow relapse of b-all in children and young adults: Childrens oncology group (Cog) study AALL1331." Pediatric Blood and Cancer 67(SUPPL 4). | Irrelevant intervention |
| Buendia, M. T., et al. (1997). "Acute lymphoblastic leukemia in children: nonrandomized comparison of conventional vs. intensive chemotherapy at the National Cancer Institute of Colombia." Med Pediatr Oncol 28(2): 108-16. | ASP vs. ASP comparison |
| Bührer, C., et al. (1990). "Central nervous system relapse prevention in 1165 standard-risk children with acute lymphoblastic leukemia in five BFM trials." Haematology and blood transfusion 33: 500-503. | Irrelevant comparator |
| Burke, M. J., et al. (2019). "Outcome in adolescent and young adult (AYA) patients compared to younger patients treated for high-risk B-lymphoblastic leukemia (HR B-ALL): report from the children's oncology group study AALL0232." Blood 134. | Irrelevant comparator |
| Cetin, M., et al. (1994). "Hyperglycemia, ketoacidosis and other complications of L-asparaginase in children with acute lymphoblastic leukemia." Journal of Medicine 25(3-4): 219-229. | Irrelevant comparator |
| Chessells, J. M., et al. (1986). "Medical Research Council leukaemia trial--UKALL V: an attempt to reduce the immunosuppressive effects of therapy in childhood acute lymphoblastic leukemia. Report to the Council by the Working Party on Leukaemia in Childhood." Journal of clinical oncology 4(12): 1758-1764. | Irrelevant comparator |
| Chessells, J. M., et al. (1995). "Intensification of treatment and survival in all children with lymphoblastic leukaemia: results of UK Medical Research Council trial UKALL X. Medical Research Council Working Party on Childhood Leukaemia." Lancet (london, england) 345(8943): 143-148. | Irrelevant comparator |
| Chessells, J. M., et al. (2002). "Failure of a new protocol to improve treatment results in paediatric lymphoblastic leukaemia: lessons from the UK Medical Research Council trials UKALL X and UKALL XI." British journal of haematology 118(2): 445-455. | Irrelevant comparator |
| Conter, V., et al. (2007). "Pulses of vincristine and dexamethasone in addition to intensive chemotherapy for children with intermediate-risk acute lymphoblastic leukaemia: a multicentre randomised trial." Lancet (london, England) 369(9556): 123-131. | Irrelevant comparator |
| Crist, W., et al. (1992). "Current results of studies of immunophenotype-, age- and leukocyte-based therapy for children with acute lymphoblastic leukemia. The Pediatric Oncology Group." Leukemia 6 Suppl 2: 162-166. | No relevant outcomes |
| Dai, Z. J., et al. (2021). "Efficacy and safety of PEG-asparaginase versus E. coli L-asparaginase in Chinese children with acute lymphoblastic leukemia: A meta-analysis." Translational Pediatrics 10(2): 244-255. | Systematic literature review |
| Teachey, D, T., et al. Cranial Radiation Can be Eliminated in Most Children with T-Cell Acute Lymphoblastic Leukemia (T-ALL) and Bortezomib Potentially Improves Survival in Children with T-Cell Lymphoblastic Lymphoma (T-LL): Results of Children's Oncology Group (COG) Trial AALL1231. ASH 2020. | Irrelevant comparator |
| de Bont JM, Holt B, Dekker AW, et al. Significant difference in outcome for adolescents with acute lymphoblastic leukemia treated on pediatric vs adult protocols in the Netherlands. Leukemia 2004;18:2032-2035. | Full text irretrievable |
| Deconinck, E., et al. (2005). "Intensive therapy before or during the conditioning regimen of allogeneic marrow transplantation in adult acute lymphoblastic leukemia patients: We must choose to reduce toxicity-A Groupe Ouest-Est d'Etude des Leucémies et Autres Maladies du Sang Study." Biology of Blood and Marrow Transplantation 11(6): 448-454. | Irrelevant comparator |
| Demiroglu, H., et al. (2020). "A comparison of allogeneic stem cell transplantation and BFM 95 chemotherapy protocol in adult patients with acute lymphoblastic leukemia." Bone marrow transplantation 55: 417-418. | No relevant outcomes |
| Domenech, C., et al. (2011). "l-asparaginase loaded red blood cells in refractory or relapsing acute lymphoblastic leukaemia in children and adults: results of the GRASPALL 2005-01 randomized trial." British Journal of Haematology 153(1): 58-65. | ASP vs. ASP comparison |
| Durrant, I. J., et al. (1997). "Intensification of treatment for adults with acute lymphoblastic leukaemia: results of U.K. Medical Research Council randomized trial UKALL XA. Medical Research Council Working Party on Leukaemia in Adults." British journal of haematology 99(1): 84-92. | Irrelevant comparator |
| Duval, M., et al. (2002). "Comparison of Escherichia coli-asparaginase with Erwinia-asparaginase in the treatment of childhood lymphoid malignancies: results of a randomized European Organisation for Research and Treatment of Cancer-Children's Leukemia Group phase 3 trial." Blood 99(8): 2734-2739. | ASP vs. ASP comparison |
| Eden, O.B., et al. (1991). "Results of Medical Research Council Childhood Leukaemia Trial UKALL VIII (report to the Medical Research Council on behalf of the Working Party on Leukaemia in Childhood)." British Journal of Haematology 78(2): 187-196. | Irrelevant comparator |
| Ekert, H., et al. (1980). "A randomized study of intermittent chemotherapy with or without BCG inoculation in maintenance therapy of childhood ALL." Medical and Pediatric Oncology 8(4): 353-360. | Irrelevant comparator |
| Erkut, N., et al. (2018). "Comparison between Hyper-CVAD and PETHEMA ALL-93 in Adult Acute Lymphoblastic Leukemia: A Single-Center Study." Chemotherapy 63(4): 207-213. | Sample size <20 per treatment arm |
| Escherich, G., et al. (2020). "Clofarabine significantly increases eradication of minimal residual disease of B-precursor all compared to high-dose cytarabine in randomized trial coall 08-09." Blood 136(SUPPL 1): 21. | Irrelevant comparator |
| Faderl, S., et al. (2000). "Outcome of philadelphia chromosome-positive adult acute lymphoblastic leukemia." Leukemia and Lymphoma 36(3-4): 263-273. | Irrelevant comparator |
| Feldges, A., et al. (1982). "[Chance of a second remission in acute juvenile lymphoblastic leukemia with favorable prognosis]." Schweiz Med Wochenschr 112(30): 1070-3. | Irrelevant comparator |
| Felice, M. S., et al. (2011). "No advantage of a rotational continuation phase in acute lymphoblastic leukemia in childhood treated with a BFM back-bone therapy." Pediatric blood & cancer 57(1): 47-55. | Irrelevant comparator |
| Fière, D., et al. (1993). "Adult acute lymphoblastic leukemia: a multicentric randomized trial testing bone marrow transplantation as postremission therapy. The French Group on Therapy for Adult Acute Lymphoblastic Leukemia." Journal of clinical oncology 11(10): 1990-2001. | Irrelevant comparator |
| Folber, F., et al. (2010). "Treatment of adult acute lymphoblastic leukemia according to GMALL 07/2003 study protocol in the Czech Republic - The first experience." Vnitrni Lekarstvi 56(3): 176-182. | Irrelevant comparator |
| Gabriele Escherich, MD, Udo Zur Stadt, PhD, Dagmar Dilloo, MD, Joerg Faber, MD PhD, Tobias Feuchtinger, MD, Thomas Imscheiler, MD, Norbert Jorch, MD, Arnulf Pekrun, Irene Schmid, MD, Martin Zimmermann, PhD, Martin A. Horstmann, MD Clofarabine Significantly Increases Eradication of Minimal Residual Disease of B-Precursor ALL Compared to High-Dose Cytarabine in Randomized Trial Coall 08-09. ASH 2020. | Irrelevant comparator |
| Gahrton, G., et al. (1974). "Induction of remission with L asparaginase, cyclophosphamide, cytosine arabinoside, and prednisolone in adult patients with acute leukemia." Cancer 34(2): 472-479. | Sample size <20 per treatment arm |
| Ganesan, P., et al. (2019). "Outcomes in adolescent and young adult (AYA) acute lymphoblastic leukemia (ALL): A report from the indian acute leukemia research database (INwARD) of the hematology cancer consortium (HCC)." Blood 134. | No relevant outcomes |
| Gavrilina, O. A., et al. (2019). "Interim results of Russian acute lymphoblastic leukemia (RALL- 2016) study with centralized MRD-monitoring and randomization for autologous HSCT with non-myeloablative conditioning in adult Ph-negative ALL patients." Blood 134. | ASP vs. ASP comparison |
| Gaynon, P. S., et al. (1988). "Intensive therapy for children with acute lymphoblastic leukaemia and unfavourable presenting features. Early conclusions of study CCG-106 by the Childrens Cancer Study Group." Lancet (london, england) 2(8617): 921-924. | Full text irretrievable |
| Gaynon, P.S., et al. (1993). "Improved therapy for children with acute lymphoblastic leukemia and unfavorable presenting features: A follow-up report of the Childrens Cancer Group study CCG-106." Journal of Clinical Oncology 11(11): 2234-2242. | ASP vs. ASP comparison |
| Goekbuget, N., et al. (2021). "First Results of the Risk-Adapted, MRD-Stratified GMALL Trial 08/2013 in 705 Adults with Newly Diagnosed Acute Lymphoblastic Leukemia/Lymphoma (ALL/LBL)." Blood 138: 362. | Irrelevant comparator |
| Gossai, N., et al. (2021). "Prognostic Impact of CNS-2 status in T-ALL: A report from the Children's Oncology Group." Journal of Clinical Oncology 39(15 SUPPL). | No relevant outcomes |
| Gottlieb, A.J., et al. (1984). "Efficacy of daunorubicin in the therapy of adult acute lymphocytic leukemia: a prospective randomized trial by cancer and leukemia group B." Blood 64(1): 267-274. | Irrelevant comparator |
| Harker-Murray, P., et al. (2021). "Intensification of Chemotherapy Using a Modified BFM Backbone for Children, Adolescents and Young Adults with T-Cell Acute Lymphoblastic Leukemia (T-ALL) and T-Cell Lymphoblastic Lymphoma (T-LL) Identifies Highly Chemorefractory Patients Who Benefit from Allogeneic Hematopoietic Stem Cell Transplantation." Blood 138: 3487. | Irrelevant comparator |
| Hayashi, R. J., et al. (2020). "Successful Outcomes of Newly Diagnosed T Lymphoblastic Lymphoma: results From Children's Oncology Group AALL0434." Journal of clinical oncology 38(26): 3062-3070. | Irrelevant comparator |
| Henze, G., et al. (1981). "The BFM 76/79 acute lymphoblastic leukemia therapy study. ERGEBNISSE DER STUDIE BFM 76/79 ZUR BEHANDLUNG DER AKUTEN LYMPHOBLASTISCHEN LEUKAMIE BEI KINDERN UND JUGENDLICHEN." KLIN. PADIATR. 193(3): 145-154. | Irrelevant comparator |
| Henze, G., et al. (1991). "Six-year experience with a comprehensive approach to the treatment of recurrent childhood acute lymphoblastic leukemia (ALL-REZ BFM 85). A relapse study of the BFM group." Blood 78(5): 1166-1172. | ASP vs ASP comparison |
| Hill, F. G., et al. (2004). "Successful treatment without cranial radiotherapy of children receiving intensified chemotherapy for acute lymphoblastic leukaemia: results of the risk-stratified randomized central nervous system treatment trial MRC UKALL XI (ISRC TN 16757172)." British journal of haematology 124(1): 33-46. | Irrelevant comparator |
| Hofmans, M., et al. (2019). "Results of successive EORTC-CLG 58 881 and 58 951 trials in paediatric T-cell acute lymphoblastic leukaemia (ALL)." British Journal of Haematology 186(5): 741-753. | ASP vs. ASP comparison |
| Hogan, L., et al. (2021). "Predictors of Severe Toxicity and Poor Efficacy of Reinduction Chemotherapy in First Relapse of B-Acute Lymphoblastic Leukemia: report from the Children's Oncology Group (COG) AALL1331." Pediatric blood & cancer 68(SUPPL 5). | Irrelevant comparator |
| Holle, L.M. (1997). "Pegaspargase: An alternative?" Annals of Pharmacotherapy 31(5): 616-624. | Systematic literature review |
| Hon, S., et al. (2019). "How we treat T-cell acute lymphoblastic leukemia(T-ALL) in adults in malaysia: A single centre experience." HemaSphere 3: 772. | No relevant outcomes |
| Hough, R., et al. (2016). "Efficacy and toxicity of a paediatric protocol in teenagers and young adults with Philadelphia chromosome negative acute lymphoblastic leukaemia: Results from UKALL 2003." British Journal of Haematology 172(3): 439-451. | ASP vs. ASP comparison |
| Huguet, F., et al. (2015). "Clofarabine for the treatment of adult acute lymphoid leukemia: The Group for Research on Adult Acute Lymphoblastic Leukemia intergroup." Leukemia and Lymphoma 56(4): 847-857. | Non-systematic review |
| Huguet, F., et al. (2018). "Intensified therapy of acute lymphoblastic leukemia in adults: Report of the randomized GRAALL-2005 clinical trial." Journal of Clinical Oncology 36(24): 2514-2523. | ASP vs. ASP comparison |
| Hunault, M., et al. (2007). "Outcome of adult T-lymphoblastic lymphoma after acute lymphoblastic leukemia-type treatment: A GOELAMS trial." Haematologica 92(12): 1623-1630. | Irrelevant comparator |
| Janka, S.G.E., et al. (1988). "First results of the CO ALL-85 cooperative study for high-risk patients with acute lymphatic leukemia." Klinische Padiatrie 200(3): 171-176. | Irrelevant comparator |
| Janka-Schaub, G. E., et al. (1996). "Randomized comparison of rotational chemotherapy in high-risk acute lymphoblastic leukaemia of childhood - Follow up after 9 years." European Journal of Pediatrics 155(8): 640-648. | Irrelevant comparator |
| Jeha, S., et al. (2019). "Improved CNS Control of Childhood Acute Lymphoblastic Leukemia Without Cranial Irradiation: st Jude Total Therapy Study 16." Journal of clinical oncology 37(35): 3377-3391. | ASP vs. ASP comparison |
| Junior, E. D. P., et al. (2015). "Prognostic factors in adolescent and adult patients with acute lymphoblastic leukemia with two protocols of chemotherapy: A cross-sectional study." Clinical Lymphoma, Myeloma and Leukemia 15(1): e7-e14. | ASP vs. ASP comparison |
| Kako, S., et al. (2016). "Meta-analysis and meta-regression analysis to compare the outcomes of chemotherapy for T- and B-lineage acute lymphoblastic leukemia (ALL): the use of dexamethasone, l-asparaginase, and/or methotrexate may improve the outcome of T-lineage ALL." Annals of Hematology 95(1): 87-92. | Systematic literature review |
| Kamps, W. A., et al. (1999). "Intensive treatment of children with acute lymphoblastic leukemia according to ALL-BFM-86 without cranial radiotherapy: results of Dutch Childhood Leukemia Study Group Protocol ALL-7 (1988-1991)." Blood 94(4): 1226-36. | ASP vs. ASP comparison |
| Kamps, W. A., et al. (2002). "BFM-oriented treatment for children with acute lymphoblastic leukemia without cranial irradiation and treatment reduction for standard risk patients: results of DCLSG protocol ALL-8 (1991-1996)." Leukemia 16(6): 1099-1111. | Irrelevant comparator |
| Karachunski, A. I., et al. (2007). "The results of a multicenter trial of acute lymphoblastic leukemia treatment on ALL-MB 91/ALL-BFM 90m in children: analysis of efficacy and toxicity." Terapevticheskii arkhiv 79(7): 19-26. | ASP vs. ASP comparison |
| Karachunskiy, A., et al. (2008). "Results of the first randomized multicentre trial on childhood acute lymphoblastic leukaemia in Russia." Leukemia 22(6): 1144-1153. | ASP vs. ASP comparison |
| Keeping, S., et al. (2021). "Heterogeneity of treatment response definitions in refractory/relapsed adult acute lymphoblastic leukemia (R/R ALL): Findings from a systematic literature review (SLR) of clinical trials." HemaSphere 5(SUPPL 2): 142. | Systematic literature review |
| Kilpatrick, R. D., et al. (2016). "Systematic review and quantitative synthesis of evidence to support regulatory review of oncaspar for treatment of Acute Lymphoblastic Leukemia (ALL)." Pharmacoepidemiology and Drug Safety 25: 525-526. | Systematic literature review |
| Kliman, D., et al. (2017). "Comparison of a pediatric-inspired treatment protocol versus standard-intensity chemotherapy for young adults with standard-risk BCR-ABL negative acute lymphoblastic leukemia." Leukemia and Lymphoma 58(4): 909-915. | ASP vs. ASP comparison |
| Koizumi, S. and Fujimoto, T. (1994). "Improvement in treatment of childhood acute lymphoblastic leukemia: a 10-year study by the Children's Cancer and Leukemia Study Group." International journal of hematology 59(2): 99-112. | No relevant outcomes |
| Kozlowski, P., et al. (2012). "High curability via intensive reinduction chemotherapy and stem cell transplantation in young adults with relapsed acute lymphoblastic leukemia in Sweden 2003-2007." Haematologica 97(9): 1414-1421. | Sample size <20 per treatment arm |
| Krishnamurthy, M.N., et al. (2020). "Randomized, Parallel Group, Open-Label Bioequivalence Trial of Intramuscular Pegaspargase in Patients with Relapsed Acute Lymphoblastic Leukemia." JCO Global Oncology (6): 1009-1016. | ASP vs. ASP comparison |
| Kurtzberg, J., et al. (2011). "Polyethylene glycol-conjugated L-asparaginase versus native L-asparaginase in combination with standard agents for children with acute lymphoblastic leukemia in second bone marrow relapse: A children's Oncology Group Study (POG 8866)." Journal of Pediatric Hematology/Oncology 33(8): 610-616. | ASP vs. ASP comparison |
| Lamanna, N., et al. (2013). "Treatment of adults with acute lymphoblastic leukemia: do the specifics of the regimen matter?: results from a prospective randomized trial." Cancer 119(6): 1186-1194. | No relevant outcomes |
| Lebedinsky, C., et al. (2016). "Systematic literature review evidence of pegaspargase for treatment of acute lymphoblastic leukemia (ALL)." Blood 128(22). | Systematic literature review |
| Lew, G., et al. (2020). "Outcomes after late bone marrow and very early central nervous system relapse of childhood B-acute lymphoblastic leukemia: A report from the Children's Oncology Group Phase III Study AALL0433." Haematologica 105(5): 46-55. | Irrelevant comparator |
| Li, M. J., et al. (2017). "Treatment for childhood acute lymphoblastic leukemia in Taiwan: taiwan Pediatric Oncology Group ALL-2002 study emphasizing optimal reinduction therapy and central nervous system preventive therapy without cranial radiation." Pediatric blood & cancer 64(2): 234-241. | Full text irretrievable |
| Liang, D. C., et al. (2010). "Long-term results of Taiwan Pediatric Oncology Group studies 1997 and 2002 for childhood acute lymphoblastic leukemia." Leukemia 24(2): 397-405. | ASP vs. ASP comparison |
| Lin, Y., et al. (2019). "Clinical efficacy and toxicity of peg-asparaginase in patients with solid tumor and all: A meta-analysis." International Journal of Clinical and Experimental Medicine 12(8): 9726-9737. | Systematic literature review |
| Locatelli, F., et al. (2017). "Protocol II vs protocol III given twice during reinduction therapy in children with medium-risk ALL." Blood 130(19): 2146-2149. | ASP vs. ASP comparison |
| Lopez-Hernandez MA, Alvarado-Ibarra M, Jimenez-Alvarado RM, et al. Adolescents with de novo acute lymphoblastic leukemia: Efficacy and safety of a pediatric vs adult treatment protocol. Gac Med Mex 2008;144: 485-489. | ASP vs. ASP comparison |
| Lynggaard, L. S., et al. (2021). "PEG-asparaginase treatment for acute lymphoblastic leukaemia in children: a network meta-analysis." Cochrane Database of Systematic Reviews (6). | Systematic literature review |
| Marks, D. I., et al. (2009). "T-cell acute lymphoblastic leukemia in adults: clinical features, immunophenotype, cytogenetics, and outcome from the large randomized prospective trial (UKALL XII/ECOG 2993)." Blood 114(25): 5136-5145. | Irrelevant comparator |
| Maury, S., et al. (2016). "Rituximab in B-Lineage Adult Acute Lymphoblastic Leukemia." New England journal of medicine 375(11): 1044-1053. | Irrelevant comparator |
| Medawar, C.V., et al. (2020). "PEG-asparaginase and native Escherichia coli L-asparaginase in acute lymphoblastic leukemia in children and adolescents: a systematic review." Hematology, Transfusion and Cell Therapy 42(1): 54-61. | Systematic literature review |
| Miller, D. R., et al. (1983). "Prognostic factors and therapy in acute lymphoblastic leukemia of childhood: CCG-141. A report from childrens cancer study group." Cancer 51(6): 1041-1049. | ASP vs. ASP comparison |
| Möricke, A., et al. (2008). "Risk-adjusted therapy of acute lymphoblastic leukemia can decrease treatment burden and improve survival: treatment results of 2169 unselected pediatric and adolescent patients enrolled in the trial ALL-BFM 95." Blood 111(9): 4477-4489. | Irrelevant comparator |
| Nachman, J. B., et al. (1998). "Augmented post-induction therapy for children with high-risk acute lymphoblastic leukemia and a slow response to initial therapy." New England Journal of Medicine 338(23): 1663-1671. | ASP vs. ASP comparison |
| Nachman, J. B., et al. (2009). "Young adults with acute lymphoblastic leukemia have an excellent outcome with chemotherapy alone and benefit from intensive postinduction treatment: A report from the Children's Oncology Group." Journal of Clinical Oncology 27(31): 5189-5194. | ASP vs. ASP comparison |
| Nesbit, M. E., et al. (1982). "Sanctuary therapy: A randomized trial of 724 children with previously untreated acute lymphoblastic leukemia. A report from Childrens Cancer Study Group." Cancer Research 42(2): 674-680. | Irrelevant comparator |
| Nookala Krishnamurthy, M., et al. (2020). "A randomized, parallel group, open label bioequivalence trial of intramuscular pegaspargase in patients with relapsed acute lymphoblastic leukemia." Pediatric blood & cancer 67 (SUPPL 4). | ASP vs. ASP comparison |
| Pathak, S., et al. (2021). "Real-World Outcomes in Adolescents and Young Adults with Acute Lymphoblastic Leukemia without Access to Allogeneic Stem Cell Transplant." Blood 138: 4373. | Sample size <20 per treatment arm |
| Parlimentary report by MRC (1971). "Treatment of acute lymphoblastic leukaemia. Comparison of immunotherapy (B.C.G.),intermittent methotrexate,and no therapy after a five-month intensive cytotoxic regimen ((Concord trial). Preliminary report to the Medical Research Council by the Leukaemia Committee and the Working Party on Leukaemia in Childhood." British Medical Journal 4(5781): 189-194. | Irrelevant comparator |
| Petersdorf, S.H., et al. (2001). "Comparison of the L10M consolidation regimen to an alternative regimen including escalating methotrexate/L-asparaginase for adult acute lymphoblastic leukemia: A Southwest Oncology Group study." Leukemia 15(2): 208-216. | ASP vs. ASP comparison |
| Pieters, R., et al. (2008). "Pharmacokinetics, pharmacodynamics, efficacy, and safety of a new recombinant asparaginase preparation in children with previously untreated acute lymphoblastic leukemia: A randomized phase 2 clinical trial." Blood 112(13): 4832-4838. | ASP vs. ASP comparison |
| Place, A.E., et al. (2015). "Intravenous pegylated asparaginase versus intramuscular native Escherichia coli L-asparaginase in newly diagnosed childhood acute lymphoblastic leukaemia (DFCI 05-001): A randomised, open-label phase 3 trial." The Lancet Oncology 16(16): 1677-1690. | ASP vs. ASP comparison |
| Ram, R., et al. (2012). "Adolescents and young adults with acute lymphoblastic leukemia have a better outcome when treated with pediatric-inspired regimens: Systematic review and meta-analysis." American Journal of Hematology 87(5): 472-478. | Systematic literature review |
| Rausen, A.R., et al. (1979). "Superiority of L-asparaginase combination chemotherapy in advanced acute lymphocytic leukemia of childhood. Randomized comparative trial of combination versus solo therapy." Cancer Clinical Trials 2(2): 137-144. | ASP vs. ASP comparison |
| Reaman, G.H., et al. (1999). "Treatment outcome and prognostic factors for infants with acute lymphoblastic leukemia treated on two consecutive trials of the Children's Cancer Group." Journal of Clinical Oncology 17(2): 445-455. | ASP vs. ASP comparison |
| Ribera, J. M., et al. (1990). "[Adult acute lymphoblastic leukemia: preliminary results of the LAL-86 protocol]." Sangre (Barc) 35(1): 26-32. | Irrelevant comparator |
| Rizzari, C., et al. (2001). "Effect of protracted high-dose L-asparaginase given as a second exposure in a Berlin-Frankfurt-Münster-based treatment: results of the randomized 9102 intermediate-risk childhood acute lymphoblastic leukemia study--a report from the Associazione Italiana Ematologia Oncologia Pediatrica." Journal of clinical oncology 19(5): 1297-1303. | ASP vs. ASP comparison |
| Rossi, M.R., et al. (1986). "Randomized multicentric Italian study on two treatment regimens for marrow relapse in childhood acute lymphoblastic leukemia." Pediatric Hematology and Oncology 3(1): 1-9. | ASP vs. ASP comparison |
| Rowntree, C. J., et al. (2021). "First Analysis of the UKALL14 Randomized Trial to Determine Whether the Addition of Nelarabine to Standard Chemotherapy Improves Event Free Survival in Adults with T-Cell Acute Lymphoblastic Leukaemia (CRUK/09/006)." Blood 138: 366. | Irrelevant comparator |
| Rytting, M. E. et al. Augmented Berlin-Frankfurt-Münster therapy in adolescents and young adults (AYAs) with acute lymphoblastic leukemia (ALL). Cancer. 2014;120(23):3660-8. | Irrelevant comparator |
| Rytting ME, Jabbour EJ, Jorgensen JL, Ravandi F, Franklin AR, Kadia TM, et al. Final results of a single institution experience with a pediatric-based regimen, the augmented Berlin–Frankfurt–Münster, in adolescents and young adults with acute lymphoblastic leukemia, and comparison to the hyper-CVAD regimen. Am J Hematol [Internet]. 2016 Aug 1 [cited 2022 Nov 17];91(8):819. Available from: /pmc/articles/PMC5558853/ | Irrelevant comparator |
| Sackmann-Muriel, F., et al. (1998). "Latin American trials in childhood acute lymphoblastic leukemia. GATLA/GLATHEM report of results from 1967 through 1994." International journal of pediatric hematology/oncology 5(2-4): 177-185. | No relevant outcomes |
| Salzer, W.L., et al. (2017). "Toxicity associated with intensive postinduction therapy incorporating clofarabine in the very high-risk stratum of patients with newly diagnosed high-risk B-lymphoblastic leukemia: a report from the Children's Oncology Group study AALL1131." Cancer (no pagination). | ASP vs. ASP comparison |
| Samarasinghe, S., et al. (2021). "Ten Year Outcomes of UKALL 2003: A Randomised Clinical Trial of Adjusting Treatment Intensity Based on Minimal Residual Disease." Blood 138: 364. | ASP vs. ASP comparison |
| Schoeman, O., et al. (2020). "PCN41 Overall Complete Remission Rate (OCRR) and Complete Remission Rate (CR) of Standard of Care (SoC) in Relapsed/Refractory (R/R) Adult Acute Lymphoblastic Leukemia (aALL): A Meta-Analysis." Value in Health 23: S428. | Systematic literature review |
| Schore, R. J., et al. (2020). "Outcomes with reduced intensity therapy in a low-risk subset of children with National Cancer Institute (NCI) standard-risk (SR) B-lymphoblastic leukemia (B-ALL): a report from Children's Oncology Group (COG) AALL0932." Journal of clinical oncology 38(15). | ASP vs. ASP comparison |
| Schrappe, M., et al. (1994). "Concept and interim result of the ALL-BFM 90 therapy study in treatment of acute lymphoblastic leukemia in children and adolescents: the significance of initial therapy response in blood and bone marrow." Klinische Padiatrie 206(4): 208-221. | No relevant outcomes |
| Schroder H, Kjeldstad M, Boesen AM, et al. Acute lymphoblastic leukaemia in Danish children and young people 10 to 19 years of age. Should young adults with acute lymphoblastic leukaemia be treated in the same way as children? Ugeskr Laeger 2006;168:2554-2558. | Full text irretrievable |
| Shu, Y. and Chang, H. (2005). "Treatment of Philadelphia chromosome-positive acute lymphoblastic leukemia in an adult." Chinese Journal of Evidence-Based Medicine 5(12): 950-954. | Systematic literature review |
| Silverman, L. B., et al. (2000). "Results of Dana-Farber Cancer Institute Consortium protocols for children with newly diagnosed acute lymphoblastic leukemia (1981-1995)." Leukemia 14(12): 2247-2256. | No relevant outcomes |
| Spoorendonk, J., et al. (2020). "PCN183 Global Incidence, Prevalence, and Survival in Relapsed/Refractory (R/R) Adult Acute Lymphoblastic Leukemia (aALL): A Systematic Literature Review (SLR)." Value in Health 23: S455. | Systematic literature review |
| Stary, J., et al. (2014). "Intensive chemotherapy for childhood acute lymphoblastic leukemia: results of the randomized intercontinental trial ALL IC-BFM 2002." Journal of clinical oncology 32(3): 174-184. | ASP vs. ASP comparison |
| Steinherz, P.G., et al. (1993). "Development of a new intensive therapy for acute lymphoblastic leukemia in children at increased risk of early relapse: The Memorial Sloan-Kettering- New York-II protocol." Cancer 72(10): 3120-3130. | Irrelevant comparator |
| Sweet, K. L., et al. (2011). "Outcomes and prognostic factors in adolescents and young adults undergoing intensive therapy for acute lymphoblastic leukemia." Blood 118(21). | Sample size <20 per treatment arm |
| Tanimoto, M., et al. (1998). "Response-oriented individualized induction therapy followed by intensive consolidation and maintenance for adult patients with acute lymphoblastic leukemia: The ALL-87 study of the Japan Adult Leukemia Study Group (JALSG)." International Journal of Hematology 68(4): 421-429. | Irrelevant comparator |
| Teik, V. L. W., et al. (2017). "Pediatric-inspired protocol improved overall survival in young adult aged 18-30 years old with philadelphia-negative acute lymphoblastic leukemia compared to the standard adult hypercvad protocol." Blood 130. | Sample size <20 per treatment arm |
| Tulstrup, M., et al. (2018). "Individualized 6-mercaptopurine increments in consolidation treatment of childhood acute lymphoblastic leukemia: a NOPHO randomized controlled trial." European journal of haematology 100(1): 53-60. | Irrelevant comparator |
| Van der Does-Van den Berg, A., et al. (1998). "Childhood acute lymphoblastic leukemia in the Netherlands." International journal of pediatric hematology/oncology 5(2-4): 125-139. | No relevant outcomes |
| Vora, A. (2004). "United Kingdom Childhood Acute Lymphoblastic Leukaemia Randomised Trial 2003 (MRC UKALL 2003)." National research register, UK. | No relevant outcomes |
| Vrooman, L. M., et al. (2021). "Efficacy and Toxicity of Pegaspargase and Calaspargase Pegol in Childhood Acute Lymphoblastic Leukemia: Results of DFCI 11-001." Journal of Clinical Oncology 39(31): 3496-3505. | ASP vs. ASP comparison |
| Wehinger, H. and Fuerste, H. O. (1981). "Intensification of induction therapy of acute lymphoblastic leukemia with L-asparaginase in children. Results of a 5-year randomised study." Klinische Padiatrie 193(3): 159-161. | Sample size <20 per treatment arm |
| Wermes, C., et al. (2000). "Asparaginase administration in children suffering from acute lymphoblastic leukemia: A systematic overview of the literature." Hamostaseologie 20(3): 151-153. | Systematic literature review |
| Widjajanto, P. H., et al. (2013). "L-asparaginase: long-term results of a randomized trial of the effect of additional 3 doses during consolidation treatment in the Indonesian WK-ALL-2000 protocol." Journal of pediatric hematology/oncology 35(8): 597-602. | ASP vs. ASP comparison |
| Wiernik, P.H., et al. (2003). "A ramdomized trial of induction therapy (daunorubicin, vincristine, prednisone versus daunorubicin, vincristine, prednisone, cytarabine and 6-thioguanine) in adult acute lymphoblastic leukemia with long-term follow-up: An Eastern Cooperative Oncology Group study (E3486)." Leukemia and Lymphoma 44(9): 1515-1521. | Irrelevant comparator |
| Winick, N., et al. (2019). "Randomized assessment of delayed intensification and two methods for parenteral methotrexate delivery in childhood B-ALL: childrens Oncology Group Studies P9904 and P9905." Leukemia. | No relevant outcomes |
| Yingying, Y., et al. (2019). "Comparison between Hypercvad and CALLG2008 Protocol in Adult Patients with Newly Diagnosed Acute Lymphoblastic Leukemia: a Single Center Study." Blood 134: 5122. | Irrelevant intervention |
| Zawitkowska, J., et al. (2018). "Clinical characteristics and analysis of treatment result in children with Ph-positive acute lymphoblastic leukaemia in Poland between 2005 and 2017." European Journal of Haematology 101(4): 542-548. | ASP vs. ASP comparison |
| Zhu (2008). "Comparison of polyethylene glycol conjugated asparaginase and L-asparaginase for treatment of childhood acute lymphoblastic leukemia." Zhonghua xue ye xue za zhi = Zhonghua xueyexue zazhi 29(1): 29-33. | ASP vs. ASP comparison |
| Zintl, F., et al. (1992). "Experiences with modified BFM protocols in the treatment of children with acute lymphoblastic leukemia (ALL) in East Germany 1981-1991." Klinische Padiatrie 204(4): 221-229. | No relevant outcomes |
| Zuna, J., et al. (2000). "Monitoring minimal residual disease in pediatric patients with acute lymphoblastic leukemia." Vnitrní lékarství 46(8): 465-469. | No relevant outcomes |

Abbreviations: ASP, asparaginase; SLR, systematic literature review.

**Table S8: Meta-analysis excluded studies at full-text screening.**

| Study | Reason for exclusion |
| --- | --- |
| Abbasi, S., et al. (2013). "Acute lymphoblastic leukemia experience: Epidemiology and outcome of two different regimens." Mediterranean Journal of Hematology and Infectious Diseases 5(1): 1-5. | Mixed Ph+/Ph- population |
| Advani, S. H., et al. (1983). "Acute lymphoblastic leukemia: End-result analysis of treatment and prognostic factors in Indian patients." American Journal of Hematology 15(1): 35-43. | No ASP vs. Hyper-CVAD comparison |
| Alabdulwahab, A. S., et al. (2017). "The Dana Farber consortium protocol (DFCP) vs. classic Hyper-CVAD for treatment of acute lymphoblastic leukemia in patients < 50 Y. Single institution experience." Leukemia Research 60: 58-62. | Mixed Ph+/Ph- population |
| Alacacioglu,I.,et al. (2014). "Is the BFM regimen feasible for the treatment of adult acute lymphoblastic Leukemia? A retrospective analysis of the outcomes of BFM and hyper-CVAD chemotherapy in two centers." Chemotherapy 60(4): 219-223. | Mixed Ph+/Ph- population |
| Amylon,M.D.,et al. (1999). "Intensive high-dose asparaginase consolidation improves survival for pediatric patients with T cell acute lymphoblastic leukemia and advanced stage lymphoblastic lymphoma: a Pediatric Oncology Group study." Leukemia 13(3): 335-342. | No ASP vs. Hyper-CVAD comparison |
| Benton, C. B., et al. (2014). "Safety and clinical activity of 5-aza-2'-deoxycytidine (decitabine) with or without Hyper-CVAD in relapsed/refractory acute lymphocytic leukaemia." British Journal of Haematology 167(3): 356-365. | No ASP vs. Hyper-CVAD comparison |
| Buyukasik, Y., et al. (2013). "Hyper-CVAD regimen in routine management of adult acute lymphoblastic leukemia: A retrospective multicenter study." Acta Haematologica 130(3): 199-205. | Mixed Ph+/Ph- population |
| Cáp, J., et al. (1983). "Treatment of acute lymphoblastic leukemia in children. Long-term results of two trials." Neoplasma 30(1): 81-92. | No ASP vs. Hyper-CVAD comparison |
| CCLSG (1989). "Treatment of childhood acute lymphoblastic leukemia: randomized trials of protocols CCLSG-L 841 and I 841. (Phase III study). Children's Cancer and Leukemia Study Group." [Rinsh ketsueki] The Japanese journal of clinical hematology 30(7): 967-974. | No ASP vs. Hyper-CVAD comparison |
| Chalandon, Y., et al. (2015). "Randomized study of reduced-intensity chemotherapy combined with imatinib in adults with Ph-positive acute lymphoblastic leukemia." Blood 125(24): 3711-3719. | No ASP vs. Hyper-CVAD comparison |
| de Diego-Floreschapa, J., et al. (1999). "[Long-term follow-up of 2 therapeutic programs for the treatment of high-risk childhood acute lymphoblastic leukemia. Experience at the 20 de Noviembre National Medical Center]." Gac Med Mex 135(3): 253-8. | No ASP vs. Hyper-CVAD comparison |
| Demichelis, R., et al. (2019). "Pediatric-inspired regimens are associated with better outcomes when compared with hypercvad in hispanic adolescents and young adults with acute lymphoblastic leukemia." Blood 134. | Earlier data from later publication |
| Dunsmore, K. P., et al. (2020). "Children's oncology group AALL0434: A phase III randomized clinical trial testing nelarabine in newly diagnosed t-cell acute lymphoblastic leukemia." Journal of Clinical Oncology 38(28): 3282-3293. | No ASP vs. Hyper-CVAD comparison |
| El-Cheikh, J., et al. (2017). "Hyper-CVAD Compared With BFM-like Chemotherapy for the Treatment of Adult Acute Lymphoblastic Leukemia. A Retrospective Single-Center Analysis." Clinical Lymphoma, Myeloma and Leukemia 17(3): 179-185. | Mixed Ph+/Ph- population |
| Fière, D., et al. (1987). "Treatment of adult acute lymphoblastic leukemia. Preliminary results of a trial from the French Group." Haematology and blood transfusion 30: 125-129. | No ASP vs. Hyper-CVAD comparison |
| Hallböök, H., et al. (2006). "Treatment outcome in young adults and children > 10 year of age with acute lymphoblastic leukemia in Sweden: A comparison between a pediatric protocol and an adult protocol." Cancer 107(7): 1551-1561. | No ASP vs. Hyper-CVAD comparison |
| Hann, I., et al. (2000). "Benefit of intensified treatment for all children with acute lymphoblastic leukaemia: results from MRC UKALL XI and MRC ALL97 randomised trials. UK Medical Research Council's Working Party on Childhood Leukaemia." Leukemia 14(3): 356-363. | No ASP vs. Hyper-CVAD comparison |
| Harris, M.B., et al. (1998). "Consolidation therapy with antimetabolite-based therapy in standard- risk acute lymphocytic leukemia of childhood: A pediatric oncology group study." Journal of Clinical Oncology 16(8): 2840-2847. | No ASP vs. Hyper-CVAD comparison |
| Herson, J., et al. (1979). "Vincristine and prednisone vs vincristine, L-asparaginase, and prednisone for second remission induction of acute lymphocytic leukemia in children." Med Pediatr Oncol 6(4): 317-23. | No ASP vs. Hyper-CVAD comparison |
| Huang, A. J., et al. (2019). "Efficacy of Hyper-CVAD/MA and CHALL-01 regimens in the treatment of Philadelphia chromosome-positive adult acute lymphoblastic leukemia patients under 60 years old." Zhonghua xue ye xue za zhi = Zhonghua xueyexue zazhi 40(8): 625-632. | No ASP vs. Hyper-CVAD comparison |
| Hutchinson, R. J., et al. (2003). "Intensification of therapy for children with lower-risk acute lymphoblastic leukemia: long-term follow-up of patients treated on Children's Cancer Group Trial 1881." Journal of clinical oncology 21(9): 1790-1797. | No ASP vs. Hyper-CVAD comparison |
| Hvizdala, E.V., et al. (1988). "Lymphoblastic lymphoma in children - A randomized trial comparing LSA2-L2 with the A-COP+ therapeutic regimen: A Pediatric Oncology Group study." Journal of Clinical Oncology 6(1): 26-33. | No ASP vs. Hyper-CVAD comparison |
| Jones, B., et al. (1977). "Optimal use of L-asparaginase (NSC-109229) in acute lymphocytic leukemia." Medical and Pediatric Oncology 3(4): 387-400. | No ASP vs. Hyper-CVAD comparison |
| Karcioglu, A.M., et al. (2018). "Pediatric chemotherapeutic regimen (BFM-95) is superior for overall survival in adult acute lymphoblastic leukemia." UHOD - Uluslararasi Hematoloji-Onkoloji Dergisi 28(4): 254-260. | Mixed Ph+/Ph- population |
| Kato, M., et al. (2014). "No impact of high-dose cytarabine and asparaginase as early intensification with intermediate-risk paediatric acute lymphoblastic leukaemia: results of randomized trial TCCSG study L99-15." British Journal of Haematology 164(3): 376-383. | No ASP vs.Hyper-CVAD comparison |
| Koller, C. A., et al. (1997). "The hyper-CVAD regimen improves outcome in relapsed acute lymphoblastic leukemia." Leukemia 11(12): 2039-2044. | No ASP vs. Hyper-CVAD comparison |
| Komp, D. M., et al. (1976). "Cyclophosphamide-asparaginase- vincristine-prednisone induction therapy in childhood acute lymphocytic and nonlymphocytic leukemia." Cancer 37(3): 1243-1247. | No ASP vs. Hyper-CVAD comparison |
| Liang, D.C., et al. (1999). "Unexpected mortality from the use of E. coli L-asparaginase during remission induction therapy for childhood acute lymphoblastic leukemia: A report from the Taiwan Pediatric Oncology Group." Leukemia 13(2): 155-160. | No ASP vs. Hyper-CVAD comparison |
| Liu, L., et al. (2015). "Efficacy of low-dose cytarabine and aclarubicin in combination with granulocyte colony-stimulating factor (CAG regimen) compared to Hyper-CVAD regimen as salvage chemotherapy in relapsed/refractory Philadelphia chromosome-negative acute lymphoblastic leukemia." Leukemia Research 39(3): 323-328. | No ASP vs. Hyper-CVAD comparison |
| Lluesma-Gonalons, M., et al. (1991). "Improved results of an intensified therapy in adult acute lymphocytic leukemia." Annals of Oncology 2(1): 33-39. | No ASP vs. Hyper-CVAD comparison |
| Locatelli, F., et al. (2020). "Superior event-free survival with blinatumomab versus chemotherapy in children with high-risk first relapse of B-cell precursor acute lymphoblastic leukemia: a randomized, controlled phase 3 trial." Bone marrow transplantation 55: 14-16. | No ASP vs. Hyper-CVAD comparison |
| Matsuzaki, A., et al. (1999). "Treatment of standard-risk acute lymphoblastic leukemia in children: the results of protocol AL841 from the Kyushu-Yamaguchi Children's Cancer Study Group in Japan." Pediatric hematology and oncology 16(3): 187-199. | No ASP vs. Hyper-CVAD comparison |
| Nagura, E., et al. (1994). "Nation-wide randomized comparative study of doxorubicin, vincristine and prednisolone combination therapy with and without L-asparaginase for adult acute lymphoblastic leukemia." Cancer Chemotherapy and Pharmacology 33(5): 359-365. | No ASP vs. Hyper-CVAD comparison |
| O'Brien, S., et al. (2008). "Results of the hyperfractionated cyclophosphamide, vincristine, doxorubicin, and dexamethasone regimen in elderly patients with acute lymphocytic leukemia." Cancer 113(8): 2097-2101. | No ASP vs. Hyper-CVAD comparison |
| Pession, A., et al. (2005). "Long-term results of a randomized trial on extended use of high dose L-asparaginase for standard risk childhood acute lymphoblastic leukemia." Journal of Clinical Oncology 23(28): 7161-7167. | No ASP vs. Hyper-CVAD comparison |
| Rytting, M. E., et al. (2014). "Augmented Berlin-Frankfurt-Münster therapy in adolescents and young adults (AYAs) with acute lymphoblastic leukemia (ALL)." Cancer 120(23): 3660-3668. | Earlier data from later publication |
| Sallan, S.E., et al. (1983). "Influence of intensive asparaginase in the treatment of childhood non-T-cell acute lymphoblastic leukemia." Cancer Research 43(11): 5601-5607. | No ASP vs. Hyper-CVAD comparison |
| Schore, R. J., et al. (2020). "Outcomes with reduced intensity therapy in a low-risk subset of children with National Cancer Institute (NCI) standard-risk (SR) B-lymphoblastic leukemia (B-ALL): a report from Children's Oncology Group (COG) AALL0932." Journal of clinical oncology 38(15). | No ASP vs. Hyper-CVAD comparison |
| Schrappe, M., et al. (2000). "Improved outcome in childhood acute lymphoblastic leukemia despite reduced use of anthracyclines and cranial radiotherapy: results of trial ALL-BFM 90. German-Austrian-Swiss ALL-BFM Study Group." Blood 95(11): 3310-3322. | No ASP vs. Hyper-CVAD comparison |
| Stryckmans, P. A., et al. (1983). "Comparison of chemotherapy with immunotherapy for maintenance of acute lymphoblastic leukemia in children and adults." Blood 62(3): 606-615. | No ASP vs. Hyper-CVAD comparison |
| Wann, S. L., et al. (2021). "Clinical outcomes of adults and young adults (AYA) with acute lymphoblastic leukemia (ALL): A multicenter analysis of pediatric-inspired protocol (MASPORE) vs hyper-CVAD in Singapore." Blood 138(SUPPL 1): 1238. | Uncertain or missing asparaginase in induction/consolidation |

Abbreviations: ASP, asparaginase; hyper-CVAD, hyper-fractionated cyclophosphamide, vincristine, doxorubicin, and dexamethasone; Ph-/Ph+, Philadelphia chromosome absent/present.
